# Updated Appropriate Use Criteria for Amyloid and Tau PET: A Report from the Alzheimer’s Association and Society for Nuclear Medicine and Molecular Imaging Workgroup

**DOI:** 10.2967/jnumed.124.268756

**Published:** 2025-06

**Authors:** Gil D. Rabinovici, David S. Knopman, Javier Arbizu, Tammie L.S. Benzinger, Kevin J. Donohoe, Oskar Hansson, Peter Herscovitch, Phillip H. Kuo, Jennifer H. Lingler, Satoshi Minoshima, Melissa E. Murray, Julie C. Price, Stephen P. Salloway, Christopher J. Weber, Maria C. Carrillo, Keith A. Johnson

**Affiliations:** 1 Department of Neurology and Department of Radiology and Biomedical Imaging, University of California at San Francisco, San Francisco, California;; 2 Mayo Clinic Neurology and Neurosurgery, Rochester, Minnesota;; 3 Department of Nuclear Medicine, University of Navarra Clinic, Pamplona, Spain;; 4 Mallinckrodt Institute of Radiology, School of Medicine, Washington University in St. Louis, St. Louis, Missouri; Knight Alzheimer’s Disease Research Center, School of Medicine, Washington University in St. Louis, St. Louis, Missouri;; 5 Nuclear Medicine, Beth Israel Deaconess Medical Center, Boston, Massachusetts;; 6 Clinical Memory Research Unit, Department of Clinical Sciences Malmö, Faculty of Medicine, Lund University, Lund, Sweden;; 7 Memory Clinic, Skåne University Hospital, Malmö, Sweden;; 8 Positron Emission Tomography Department, National Institutes of Health Clinical Center, Bethesda, Maryland;; 9 Medical Imaging, Medicine, and Biomedical Engineering, University of Arizona, Tucson, Arizona;; 10 Department of Health and Community Systems, University of Pittsburgh, Pittsburgh, Pennsylvania;; 11 Department of Radiology and Imaging Sciences, University of Utah, Salt Lake City, Utah;; 12 Department of Neuroscience, Mayo Clinic, Jacksonville, Florida;; 13 Department of Radiology, Massachusetts General Hospital, Boston, Massachusetts;; 14 Department of Neurology and Psychiatry the Warren Alpert School of Medicine, Brown University, Providence, Rhode Island;; 15 Butler Hospital Memory and Aging Program, Providence, Rhode Island;; 16 Alzheimer’s Association, Chicago, Illinois;; 17 Center for Alzheimer Research and Treatment, Department of Neurology, Brigham and Women’s Hospital, Boston, Massachusetts;; 18 Molecular Neuroimaging, Massachusetts General Hospital, Boston, Massachusetts;; 19 Harvard Medical School, Boston, Massachusetts; and; 20 Departments of Neurology and Radiology, Massachusetts General Hospital, Boston, Massachusetts

**Keywords:** amyloid PET, tau PET, Alzheimer disease, diagnosis, clinical care, treatment, neuroimaging, biomarkers, cognitive impairment, dementia, PET, appropriate use criteria, molecular imaging, neurology, memory disorders, early detection, therapeutic strategies, PET imaging, brain pathology

## Abstract

The Alzheimer’s Association and the Society of Nuclear Medicine and Molecular Imaging convened a multidisciplinary workgroup to update appropriate use criteria (AUC) for amyloid positron emission tomography (PET) and to develop AUC for tau PET. **Methods:** The workgroup identified key research questions that guided a systematic literature review on clinical amyloid/tau PET. Building on this review, the workgroup developed 17 clinical scenarios in which amyloid or tau PET may be considered. A modified Delphi approach was used to rate each scenario by consensus as “rarely appropriate,” “uncertain,” or “appropriate.” Ratings were performed separately for amyloid and tau PET as stand-alone modalities. **Results:** For amyloid PET, 7 scenarios were rated as appropriate, 2 as uncertain, and 8 as rarely appropriate. For tau PET, 5 scenarios were rated as appropriate, 6 as uncertain, and 6 as rarely appropriate. **Conclusion:** AUC for amyloid and tau PET provide expert recommendations for clinical use of these technologies in the evolving landscape of diagnostics and therapeutics for Alzheimer’s disease.

## HIGHLIGHTS

- A multidisciplinary workgroup convened by the Alzheimer’s Association and the Society of Nuclear Medicine and Molecular Imaging updated the appropriate use criteria (AUC) for amyloid PET and to develop AUC for tau PET.
- The goal of these updated AUC is to assist clinicians in identifying clinical scenarios in which amyloid or tau PET may be useful for guiding the diagnosis and management of patients who have, or are at risk for, cognitive decline.
- These updated AUC are intended for dementia specialists who spend a significant proportion of their clinical effort caring for patients with cognitive complaints, as well as serve as a general reference for a broader audience interested in implementation of amyloid and tau PET in clinical practice.

## 1. INTRODUCTION AND SCOPE

Alzheimer’s disease (AD) is defined neuropathologically by the deposition of extracellular plaques composed of aggregated forms of the amyloid-beta (Aβ) polypeptide and intraneuronal neurofibrillary tangles (NFTs) composed of aggregated hyperphosphorylated tau protein (1*,*^2^). In the past 20 years, positron emission tomography (PET) radiotracers have been developed to image amyloid plaques and tau tangles in vivo (3–9). Currently, 3 fluorine-18-labeled amyloid radiotracers (^18^F-florbetapir, ^18^F-flutemetamol, ^18^F-florbetaben) are approved for clinical use by regulatory agencies in the U.S., the European Union, and other countries to estimate amyloid plaque density in adult patients with cognitive impairment who are being evaluated for AD and other causes of cognitive decline. In 2020, the U.S. Food and Drug Administration (FDA) approved the tau radiotracer ^18^F-flortaucipir (FTP) (10) to estimate the density and distribution of NFTs in adult patients with cognitive impairment who are being evaluated for AD.

**RESEARCH IN CONTEXT**

**SYSTEMATIC REVIEW:** The Alzheimer’s Association and the Society of Nuclear Medicine and Molecular Imaging convened a multidisciplinary workgroup to update appropriate use criteria (AUC) for amyloid PET and to develop AUC for tau PET. Oregon Health & Science University conducted a systematic review of the literature to summarize and assess the strength of evidence for the safety, diagnostic accuracy, and effect on patient outcomes of amyloid and tau PET.

**INTERPRETATION:** AUC for amyloid and tau PET provide expert recommendations for clinical use of these technologies in the evolving landscape of diagnostics and therapeutics for Alzheimer disease.

**FUTURE DIRECTIONS:** Additional work is needed to integrate amyloid and tau PET into diagnostic algorithms and clinical practice guidelines that consider the broader, growing landscape of molecular biomarkers of AD pathophysiology.

In 2013, a task force convened by the Alzheimer’s Association (AA) and the Society of Nuclear Medicine and Molecular Imaging (SNMMI) developed appropriate use criteria (AUC) to define the types of patients and clinical circumstances in which amyloid PET could be used and, equally important, the clinical scenarios in which amyloid PET was felt to be inappropriate (11). The goal of this article is to update the AUC for amyloid PET from the additional data that have emerged in the decade since the original AUC were published, which include advances in therapeutics designed to lower the cerebral amyloid burden. Recognizing these important advances, in October 2023, the U.S. Centers for Medicare and Medicaid Services (CMS) retired its 2013 National Coverage Decision, which restricted coverage of amyloid PET to a single scan per patient under approved research studies, thus promoting greater patient access to this important clinical tool. CMS did not issue a noncoverage policy for tau PET; thus, it is covered by CMS under the discretion of the local Medicare Administrative Contractors. In addition, we propose for the first time AUC for tau PET, recognizing that this is a relatively novel technology and that data on its clinical utility are currently limited. The revised AUC were developed by a multidisciplinary workgroup of experts convened by AA-SNMMI (see Section 7: Methods).

The primary goal of these updated AUC is to assist clinicians in identifying clinical scenarios in which amyloid or tau PET may be useful for guiding the diagnosis and management of patients who have, or are at risk for, cognitive decline, while also highlighting scenarios in which PET scans are unlikely to provide clinically useful information. The primary intended audience is dementia specialists who spend a significant proportion of their clinical effort caring for patients with cognitive complaints. The article is also meant to serve as a general reference for a broader audience interested in implementation of amyloid and tau PET in clinical practice. In addition, the AUC are intended to support policy makers and payers in promoting cost-effective access to this important diagnostic tool to patients who are most likely to benefit in the setting of limited healthcare resources. Finally, the workgroup members recognize that amyloid and tau PET are part of a growing landscape of molecular biomarkers of AD pathophysiology, which include cerebrospinal fluid (CSF) and blood-based biomarkers of amyloid, tau, and neurodegeneration. The reader is referred to published AUC for CSF biomarkers (12) and appropriate use recommendations (AURs) for blood-based AD biomarkers (13). The optimal integration of the entire armamentarium of AD biomarkers into future diagnostic and care algorithms is beyond the scope of this article but represents an important area for future research.

## 2. BACKGROUND

The current document is an update of the previously published AUC for amyloid PET (11). The update integrates extensive literature published over the past decade that examined the diagnostic and prognostic value of amyloid PET in longitudinal clinical cohorts and observational studies; evaluated the clinical utility of amyloid PET for patient diagnosis, management, and health outcomes; further validated the diagnostic validity of amyloid PET in prospective PET-to-autopsy studies; and used amyloid PET in AD clinical trials, including for the development of amyloid-targeting antibodies that recently received approval from the U.S. FDA for the treatment of early clinical stages of AD (14–16). The updated AUC reflect an increasing awareness that amyloid deposition begins 2 decades or more before the onset of cognitive impairment, defining a prolonged preclinical phase of AD, with potential increased demand for testing among cognitively unimpaired (CU) individuals or individuals experiencing subjective cognitive decline (SCD; see Section 3: Key Definitions). The updated AUC also examine for the first time the potential role of tau PET in common clinical scenarios, given recent FDA approval of ^18^F-FTP for clinical use. An important observation is that the neocortical tau PET signal appears more proximally to clinical symptoms than does the neocortical amyloid PET signal. In contrast to the much more extensive literature on amyloid PET, ^18^F-FTP is a relatively new radiopharmaceutical with limited data, in particular as it pertains to longitudinal follow-up and clinical utility. As with amyloid imaging, recommendations represent expert opinion based on currently available information.

Amyloid and tau PET detects amyloid plaques and NFTs, the core elements that collectively define AD neuropathology. In the clinical setting, the primary role of these scans is to provide evidence for or against the presence of these disease-defining lesions in patients who are seeking assessment for cognitive symptoms. The PET scans should be performed when there is significant uncertainty regarding the etiology of cognitive impairment after a comprehensive assessment by a dementia specialist (see Section 3: Key Definitions), when AD is a diagnostic consideration, and when knowledge of amyloid or tau status is expected to help establish an etiological diagnosis and guide patient management (e.g., to confirm the presence of amyloid plaques in a patient who is a candidate for amyloid-lowering therapy). Amyloid or tau PET should not be used as a substitute for a comprehensive clinical examination, which should include a detailed medical and neurobehavioral history, physical examination, mental status testing, blood tests to rule out potentially reversible causes of cognitive impairment, and structural brain imaging. The entirety of these clinical data are required to optimally integrate amyloid/tau PET results into clinical decision making regarding diagnosis and patient management.

The guidelines presented here highlight general principles for integrating amyloid and tau PET into clinical care, including the potential appropriateness of testing in specific clinical scenarios. These guidelines represent general recommendations and should not be considered a substitute for clinical judgment exercised by the healthcare provider caring for an individual patient.

As recommended in the previous AUC, the following sequence of events would generally be appropriate for the integration of amyloid or tau PET into clinical practice: (1) evaluation by a dementia expert to assess the need for diagnostic testing, possibly to include amyloid or tau PET, if the AUC are met; (2) referral to a qualified provider of PET services; (3) performance, interpretation, and reporting of the PET result according to established standards; (4) incorporation of the PET result into the clinical assessment process by the dementia expert; and (5) disclosure of the PET result by the dementia expert to the patient, family, and care partners, along with discussion of the result and its management consequences.

## 3. KEY DEFINITIONS

The following definitions provide clarification of key terms used in this document and the clinical scenarios for appropriate use presented by this workgroup.

### 3.1 The Continuum of Cognitively Unimpaired, Subjective Cognitive Decline, Mild Cognitive Impairment, and Dementia

Cognitive impairment acquired in adulthood is diagnosed by a history from the patient and a knowledgeable proxy for the patient and by examination of objective cognitive performance under direct observation by a skilled clinician. Cognitive functioning exists on a continuum anchored at one end by the state of being cognitively unimpaired and, on the other end, by the state of severe dementia, with intermediate states in between. The definitions of cognitive impairment to be used in the current document are grounded in the clinical judgment that they represent a decline from a prior higher level of functioning. More detailed definitions are found in the National Institute on Aging and Alzheimer’s Association (NIA-AA) Research Framework consensus definitions (Table 5 in (17)) and the revised criteria for diagnosis and staging of Alzheimer’s disease (18), but the following definitions are used by this workgroup to establish AUC for amyloid and tau PET.

- **Cognitively unimpaired (CU):** Cognitive performance is within the expected range for that individual based on clinical judgment or cognitive test performance, and the patient does not endorse significant cognitive complaints (17).
- **Subjective cognitive decline (SCD):** Cognitive complaints in the absence of objective evidence of decline below expected normative levels (19).
- **Mild cognitive impairment (MCI):** Cognitive performance in at least 1 domain that is below the expected range for that individual based on all available information, but daily activities are performed in a largely independent manner. The definition of MCI allows for mild functional impact on the more complex activities of daily life (17*,*^20^).
- **Dementia:** Substantial cognitive impairment that affects multiple cognitive domains, interferes with daily functioning, and results in loss of independence. Dementia can be further subdivided into mild, moderate, and severe stages, reflecting incrementally worse functioning first in instrumental (i.e., complex) and then in basic activities of daily living (17*,*^21^).

Clinical diagnosis requires the use of categorical syndromic diagnostic labels such as SCD, MCI, or dementia, but there are many patients whose clinical presentation falls in between 2 of these labels. Thus, although this document makes recommendations that are syndrome specific, clinical judgment requires that each patient be understood as unique and not as a generic exemplar of a categorical diagnosis.

### 3.2 AD and the Etiology of Cognitive Disorders

In the context of the current document, in which amyloid and tau biomarkers are being applied to patients with cognitive impairment, we maintain a conceptual separation between cognitive disorders and underlying etiology. The most common symptomatic presentation of AD pathology is a disorder that begins with amnestic complaints that may not substantially interfere with daily activities, and then progresses to a multidomain cognitive disorder (i.e., variably involving language, visuospatial and executive deficits, as well as behavioral abnormalities) (20*,*^21^). The clinical syndrome of amnestic dementia, originally referred to as probable AD in the 1984 National Institute of Neurologic and Communicative Disorders and Stroke and the Alzheimer Disease and Related Disorders Association (NINCDS-ADRDA) criteria (22), is often, but not always, due to AD pathology. Neuropathologic investigations (23) have shown that clinical diagnostic criteria alone have suboptimal accuracy for AD as defined pathologically. Moreover, several non-amnestic cognitive presentations that are more common in younger patients, such as visual, language, or behavioral/dysexecutive variants, were shown to be due to AD neuropathology (24). The lack of a close clinical-pathologic relationship between clinical presentation and neuropathologic (or biomarker) evidence for AD requires us to recognize the pleomorphic clinical presentations of AD pathology, and that in the setting of historically typical amnestic cognitive disorders, alternative brain pathologies could be relevant.

### 3.3 Cognitive Disorder of Uncertain Etiology

We define “cognitive disorder of uncertain etiology” in this document (which is explicitly AD centric) as being present when there are simultaneously features that are typical for AD pathology and features that are typical for non-AD pathology. In the 1984 NINCDS-ADRDA criteria (22), this pattern of features that did not exclude AD but were not specific for AD was assigned a diagnosis of “possible AD.” Prior to amyloid PET (11), such symptom complexes were labeled as “unexplained.” Advances in neuropathology and antemortem biomarker investigations have shed new light on this common situation. First, many clinical features – cognitive symptoms, noncognitive symptoms, temporal profile, associated medical diagnoses, structural imaging features – are not as specific for one diagnosis as previously believed. Further, multi-etiological cognitive disorders are more common than single etiological disorders (25), so that striving to apply one and only one etiological diagnosis is conceptually naïve. Although such a group of possible AD and unexplained MCI or dementia represents a heterogeneous group, it is an important group for the current discussion of AUC for amyloid and tau PET.

### 3.4 Dementia Expert

The appropriate integration of amyloid and tau PET into the assessment of cognitive decline requires clinical expertise and experience in the evaluation of dementia. Consistent with previous AUC (11), we define a “dementia expert” as a physician typically trained and board-certified in neurology, psychiatry, or geriatric medicine who devotes a substantial proportion (at least 25%) of patient contact time to the evaluation and care of adults with acquired cognitive impairment or dementia. Physicians can self-identify as a dementia expert based on their training, knowledge base, and clinical experience. Not all neurologists, psychiatrists, or geriatricians are dementia experts; conversely, clinicians trained in other disciplines may possess the requisite expertise in dementia care. The guiding principles are that dementia experts should be (1) skilled at evaluating, diagnosing, and staging a broad spectrum of cognitive disorders; (2) familiar with the techniques of amyloid and tau PET (including their strengths and limitations); (3) able to interpret the meaning of amyloid and tau PET results in the broader clinical context of individual patients; and (4) able to communicate PET results and their implications for diagnosis and care to patients and families in a safe and effective manner, using best practices for disclosure. As clinical applications of amyloid and tau PET become more pervasive, it is likely that a broader cohort of clinicians will develop the expertise necessary to incorporate these tools into their diagnostic workup.

## 4. AMYLOID PET AND TAU PET TECHNOLOGY, RADIOTRACERS, AND INTERPRETATION

This section complements and updates information provided in the 2013 publication on the AUC for amyloid PET (11). PET is an established molecular imaging technique that is used to detect, measure, and map molecular targets in the living human, which includes being used for the in vivo localization of aggregated proteins, such as amyloid plaques and tau NFTs. Localization is possible because PET can measure the in vivo distribution of radioactive positron-emitting imaging agents, or radiopharmaceuticals, that bind selectively and specifically to the protein target. The high sensitivity of PET enables measurement of picomolar in vivo concentrations after intravenous administration of trace amounts of the radiopharmaceutical (or radioligand). In studies of neurodegeneration, carbon-11 and fluorine-18 are the positron-emitting radionuclides that are most often incorporated into pharmaceuticals, yielding radiopharmaceuticals with radioactive half-lives of about 20 minutes and 110 minutes, respectively. The longer half-life of fluorine-18 enables widespread distribution and use of these radiopharmaceuticals beyond the manufacturing site.

**FIGURE 1.**
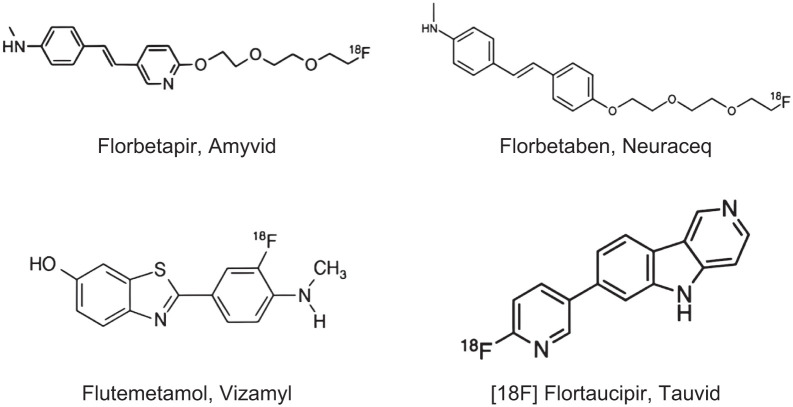
Chemical structures of amyloid and tau radiotracers.

Carbon-11 Pittsburgh compound-B (PiB) is a well-established radiopharmaceutical (26) that is widely used by research groups that can produce it on-site. PiB often serves as a reference standard to which other amyloid PET agents are compared. Three fluorine-18 Aβ agents are approved by the U.S. FDA, European Medicines Agency, and other global regulatory agencies for clinical use “to estimate β-amyloid neuritic plaque density in adult patients with cognitive impairment who are being evaluated for Alzheimer Disease (AD) and other causes of cognitive decline” (27–29): ^18^F-florbetapir (commercial name Amyvid), ^18^F-florbetaben (Neuraceq), and ^18^F-flutemetamol (Vizamyl). A fourth ^18^F-labeled agent, ^18^F-flutafuranol (formerly NAV4694) (8), is currently under clinical development, although it is not currently approved for use in the U.S. or Europe. Figure 1 illustrates the chemical structures of the FDA-approved amyloid tracers and tau tracer (Tauvid) (10*,*^27^–^30^), and Table 1 describes their use in more detail. The reader is referred to the SNMMI Procedure Standard/European Association of Nuclear Medicine (EANM) Practice Guideline for Amyloid PET Imaging of the Brain (31) for more information on how to perform an amyloid PET scan.

**TABLE 1.** FDA-Approved Diagnostic Agents

Agent	Amount	Image display	Number of regions for a positive scan
**Amyloid**			
Florbetapir F-18	370 MBq (10 mCi)	Color scale: gray scale or inverse gray scale	2, or only 1 if gray matter uptake exceeds white matter uptake
		Regions: temporal, parietal (including precuneus), frontal, and occipital	
Flutemetamol F-18	185 MBq (5 mCi)	Color scale: rainbow or Sokoloff; color scale is adjusted to set pons to approximately 90% maximum intensity	1
		Regions: temporal, parietal, posterior cingulate/precuneus, frontal, striatum	
Florbetaben F-18	300 MBq (8.1 mCi)	Color scale: gray scale or inverse gray scale	1
		Regions: temporal, parietal, posterior cingulate/precuneus, and frontal	
**Tau**			
Flortaucipir F-18	370 MBq (10 mCi)	Color scale: color scale with rapid transition between 2 distinct colors, scale being adjusted so that transition occurs at 1.65-fold threshold; neocortical activity in either hemisphere contributes to image interpretation	Positive scan shows increased neocortical activity in posterolateral temporal, occipital, or parietal/precuneus region(s), with or without frontal activity; neocortical activity in either hemisphere can contribute to identification of positive pattern (31,32)

**FIGURE 2.**
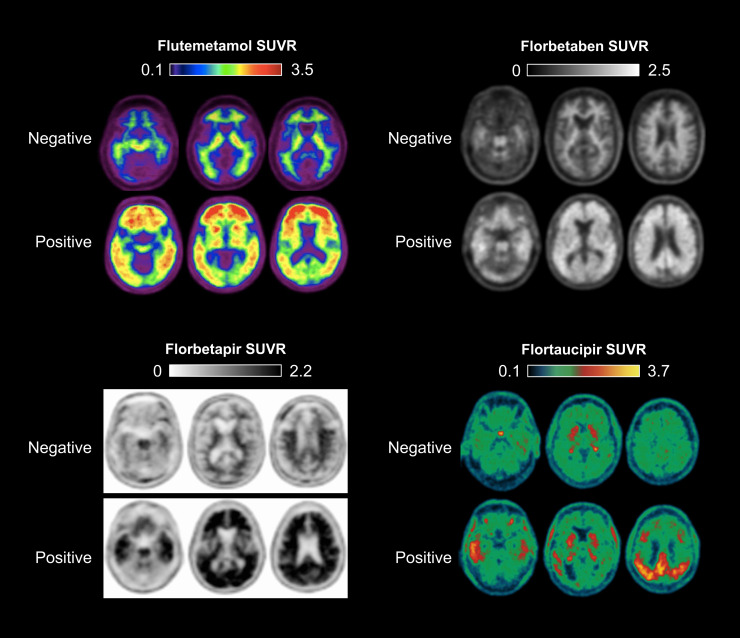
Examples of positive and negative Aβ and tau PET scans with FDA-approved radiotracers. SUVR images were created using pons (^18^F-flutemetamol), whole cerebellum (^18^F-florbetaben, ^18^F-florbetapir), and inferior cerebellar gray matter (^18^F-flortaucipir) as reference regions. Each image is displayed in approved gray and white or color scale for clinical interpretation.

The clinical interpretation of amyloid PET scans is based primarily on visual interpretation methods approved by regulatory agencies following validation in PET-to-autopsy studies performed in end-of-life populations (32–34). In patients with absent-to-low density of amyloid plaque deposition, PET scans show only nonspecific tracer retention in white matter. In patients with moderate-to-high density of amyloid plaques, tracer retention extends into the neocortex (Figure 2). The earliest amyloid PET signal is often seen in the posterior cingulate cortex, precuneus, and frontal regions (35), and widespread neocortical uptake is common by the time patients develop cognitive impairment. Each of the 3 FDA-approved amyloid radiotracers is visualized in different gray/white or color scales (Figure 2), and the specific criteria for scan positivity (including the specific regions investigated) differ slightly across the 3 agents (36).

Quantification of amyloid PET is often performed in research studies and clinical trials. The most common quantitative measure is the standardized uptake value ratio (SUVR), which is the ratio of radiopharmaceutical uptake in a target region (e.g., neocortical regions that are known to accumulate amyloid plaques) divided by uptake in a nonspecific reference region that is relatively spared of pathology (e.g., cerebellum), measured at a time after injection when these ratios were shown to be stable (varies by radiotracer). The “Centiloid” scale can be used to standardize and compare amyloid PET quantification across radiotracers and image processing methods. In this scale, 0 Centiloids (CL) represents the average neocortical uptake in young CU individuals who are unlikely to have amyloid deposition, whereas 100 CL represents the mean uptake in patients with mild-moderate dementia due to AD. Thresholds for scan positivity typically vary between 10 and 40 CL units, with lower thresholds increasing the sensitivity to detect early pathology (37–39). Standardized imaging acquisition and processing is established for amyloid PET, and several commercial software packages that can be used to derive SUVR and CL outcomes have been developed to assist with scan interpretation in clinical practice. Quantification is not currently included in the FDA labels (40), although it has been added as an adjunct to visual inspection for all 3 amyloid radiotracers in Europe. Future clinical use of amyloid PET quantification may be particularly important for objectively gauging longitudinal changes in amyloid burden in individual patients, for example, to measure clinical response to an amyloid-lowering therapy (see Section 8.3: Rationale for Clinical Scenario Appropriateness Ratings, Clinical Scenario 15) (41).

Tau PET is currently performed by using F-18 radiopharmaceuticals. ^18^F-FTP (commercial name: Tauvid) was the first widely used tau agent, and in 2020 was granted FDA approval “to estimate the density and distribution of aggregated tau NFTs for adult patients with cognitive impairment who are being evaluated for Alzheimer’s disease” (10*,*^42^).

Several additional tau-selective radiotracers were subsequently developed, including ^18^F-MK-6240, ^18^F-RO948, ^18^F-GTP-1, ^18^F-PI-2620, and ^18^F-PM-PBB3 (also known as ^18^F-APN-1607), although none have yet received FDA approval (43). All tau tracers were developed based on their ability to bind to AD-related NFTs. Most show absent-to-weak binding to non-AD tauopathies (e.g., progressive supranuclear palsy [PSP], corticobasal degeneration [CBD], chronic traumatic encephalopathy, molecular subtypes of frontotemporal dementia [FTD]), although ^18^F-PI-2620 and ^18^F-PM-PBB3 are currently being evaluated as broader spectrum tau imaging agents. Notably, ^18^F-PI2620 received orphan drug indication as a biomarker for tau deposition in 4-repeat tauopathies (i.e., PSP and CBD). All tau tracers exhibit varying degrees and patterns of “off-target” binding (i.e., binding to non-tau targets), typically in the basal ganglia, meninges, choroid plexus, and midbrain nuclei (substantia nigra and red nucleus).

As with amyloid tracers, clinical interpretation of FTP tau PET scans is based on visual interpretation (Figure 2). A scan is interpreted as showing a “negative AD tau pattern” if there is no neocortical tracer uptake, or if uptake is limited to the medial temporal, anterolateral temporal, or frontal cortex. A “positive AD pattern” is defined as showing the extension of tracer retention into the posterolateral temporal or occipital cortex, with further extension into the parietal cortex, posterior cingulate/precuneus cortex, and frontal cortex seen in more advanced disease (Figure 2) (10). In research studies, SUVR values are calculated to quantify tau PET uptake across radiotracers in various target regions of interest, with the earliest signal typically detectable in the entorhinal cortex and other medial temporal structures, followed by spread into the inferior temporal gyrus (the latter usually occurring in the setting of a positive amyloid PET scan). Efforts are underway to develop standardized quantitative tau PET scales across radiotracers and analytic approaches, analogous to the CL scale used for amyloid PET standardization (44*,*^45^). Tau PET quantification may enhance sensitivity for early-stage disease (e.g., Braak stages III/IV) (46), assist with disease staging (16*,*^18^), and gauge longitudinal change in tau burden as a result of disease progression or in response to therapeutic interventions (47).

Standardized acquisition of the PET scans, following FDA labels, is necessary for reproducible results. All nuclear medicine examinations should be performed under the supervision of and interpreted by a physician certified in nuclear medicine or nuclear radiology by the American Board of Nuclear Medicine or the American Board of Radiology in the U.S. or equivalent organizations outside the U.S. The clinical value of amyloid/tau PET imaging is entirely dependent on the quality of the images and the accuracy of interpretation. Amyloid and tau PET imaging are technically challenging and should be performed only when there is strict attention to quality control. Clinical PET scanning is widely available, but the experience of PET facilities with brain imaging is variable. Amyloid and tau imaging are evolving modalities; therefore, image interpretation criteria, the clinical significance of positive and negative scan results, and technical imaging considerations are evolving. The following recommendations are based on current knowledge and may require modification in the future. The individual performing the scan must be familiar with brain anatomy and have adequate specific training in amyloid PET interpretation. Training specific to the interpretation of amyloid imaging such as provided by the manufacture of the radiopharmaceutical (if available) should be completed and preferably augmented by training programs offered by professional societies such as the SNMMI and the EANM. High-quality training of readers is essential to ensure consistently accurate interpretation of amyloid and tau PET results. As with all nuclear medicine imaging, readers also need to learn to recognize important technical or patient-related artifacts (40).

Imaging procedures should be performed by a qualified nuclear medicine technologist with appropriate training and certification. All nuclear medicine examinations should be performed by a qualified nuclear medicine technologist who is registered/certified in nuclear medicine by the Nuclear Medicine Technology Certification Board, the American Registry of Radiologic Technologists, or equivalent organizations outside the U.S. The nuclear medicine technologist works under the supervision of a physician with qualifications outlined earlier. Imaging should be performed in an imaging facility certified by the Intersocietal Commission for the Accreditation of Nuclear Laboratories, the American College of Radiology, or other equivalent accrediting agency.

Results of amyloid PET imaging should be communicated to the referring physician by the imaging physician by way of a written report according to a standard diagnostic imaging practice as outlined in the SNMMI General Imaging Guideline. The final reading should conform to radiotracer-specific criteria for elevated amyloid or tau levels. Indeterminate results may arise due to technical or physiological factors and should be reported as such. The report should not confound amyloid/tau positivity with cognitive impairment due to AD. The dementia specialist should then communicate with patients and family members after a comprehensive review of the clinical assessment and test results.

## 5. NEUROPATHOLOGICAL TARGET OF AMYLOID AND TAU PET LIGANDS

At autopsy, amyloid plaques are visualized by using thioflavin fluorescent dyes, silver impregnation techniques, or antibody-based immunohistochemistry. Neuritic plaques are the pathognomonic plaque type in AD that are morphologically defined by the incorporation of dystrophic tau-positive neurites into the amyloid deposit (1*,*^2^). The topographic distributions of amyloid plaque deposition and NFT accumulation are used to assess the level of AD neuropathological change (ADNC), as reflected by the “ABC” score in the NIA-AA neuropathological guidelines (1*,*^2^): The Amyloid component is derived from the topographic distribution of any plaque type using the Thal amyloid phase (48); the tau component relies on the Braak tangle stage (49*,*^50^); and, given the significance of neuritic plaques, an additional amyloid component is accounted for by the Consortium to Establish a Registry for Alzheimer’s Disease (CERAD) score (51). The ABC score integrates all 3 components in order to classify an individual as having “no,” “low,” “intermediate,” or “high” ADNC, with “intermediate-high” changes considered clinically relevant (1*,*^2^).

Neuroimaging and neuropathology studies demonstrate common spatial patterns of amyloid deposition that begin in the neocortex, next involve limbic structures and the diencephalon, and lastly occur in the cerebellum (35*,*^48^*,*^52^–^54^). The topographic distribution of amyloid plaques is similar across different clinical presentations of AD (i.e., memory-, dysexecutive-, language-, and visuospatial-predominant presentations) (55–57).

In typical AD, tau accumulation is first observed in the entorhinal cortex (Braak stages I–II), followed sequentially by involvement of limbic and paralimbic structures (Braak stages III–IV) and association cortices (Braak stage V), and lastly primary cortices (i.e., primary sensorimotor, visual, or auditory cortices, Braak stage VI) (49*,*^50^). Less commonly, the distribution of tangles presents instead with “hippocampal-sparing” or “limbic-predominant” patterns. Hippocampal-sparing AD is defined by greater cortical involvement relative to limbic structures and is more commonly observed in patients presenting with an atypical, non-amnestic phenotype (24*,*^58^). In direct contrast, limbic structures are greatly affected relative to the cortex in limbic-predominant AD, with the overwhelming majority of patients presenting with an amnestic phenotype. Different clinical variants of AD show distinct topographic densities of NFTs, with the highest tangle densities found in the regions that are most clinically affected (59). Studies with tau PET have replicated these 3 patterns of tau distribution in vivo (60*,*^61^).

FDA approvals of amyloid and tau PET radiotracers (and European Medicines Agency approval of amyloid PET radiotracers) were based on studies that compared visual interpretation of antemortem PET to the distribution of amyloid and tau aggregates at autopsy. The pivotal studies leading to regulatory approval were conducted in participants near the end of life, resulting in short (several months) intervals between PET and autopsy (32–34). For amyloid tracers, the majority of visual reads of amyloid PET scans conducted with FDA-approved radiotracers were found to have 88%–98% sensitivity and 80%–95% specificity when compared with CERAD moderate-frequent neuritic plaques at autopsy. Studies that compared antemortem PET to Thal phase found that scan positivity typically corresponded to Thal phase 2–3 (62). Thus, it is important to note that a negative scan does not equate to “no” amyloid deposition, although low levels of amyloid that are below the threshold of detection are much less likely to contribute to cognitive impairment (63). Conversely, positive scan results can be seen in patients who have diffuse amyloid plaque deposition (often seen in diffuse Lewy body disease) or cerebrovascular amyloid deposits (in cerebral amyloid angiopathy), but who do not meet the neuropathological criteria for intermediate-high ADNC (64*,*^65^).

In the autopsy validation study of ^18^F-FTP (10), the majority of visual reads of antemortem PET scans showed 92% sensitivity and 80% specificity when compared with Braak stage ≥ V neurofibrillary pathology. This degree of tau neuropathology is nearly always associated with cognitive impairment and amyloid PET positivity. Therefore, a positive visual read of ^18^F-FTP PET in isolation may be sufficient to rule in a significant contribution of AD to cognitive impairment. However, when the visual read method described earlier was applied, scans were visually read as consistent with AD in only ∼20% of patients who died with Braak stage III–IV tau pathology, although this level represents the median Braak stage observed in patients who died at the MCI stage of impairment. Quantification of tau PET, in particular in the medial temporal lobe, may enhance the sensitivity of the scan to earlier Braak stages (46), but this is not performed routinely in clinical practice. The limited sensitivity of ^18^F-FTP PET to early-stage disease due to the visual read method used in the autopsy validation study may limit the clinical utility of the scan in patients with MCI or earlier clinical stages that are typically associated with less advanced tau pathology.

## 6. RELATION OF AMYLOID AND TAU PET TO OTHER DIAGNOSTICS

### 6.1 Other Nuclear Medicine Procedures

Positron emission tomography with the radiolabeled glucose analog ^18^F-fluorodeoxyglucose (FDG) has been used to image regional cerebral glucose metabolism in a wide variety of neuropsychiatric diseases for over 4 decades. ^18^F-FDG PET can be helpful in the differential diagnosis of cognitive disorders by demonstrating characteristic patterns of glucose hypometabolism that are uniquely associated with characteristic underlying neuropathologies. The most common ^18^F-FDG pattern in AD reveals hypometabolism in the temporoparietal cortex, with prominent involvement of the posterior cingulate cortex and precuneus. The frontal cortex is typically spared in early clinical stages. The anatomical pattern overlaps to a large extent with cortical atrophy seen on magnetic resonance imaging (MRI), but some studies suggest that ^18^F-FDG may be more sensitive than MRI at early disease stages, and patterns may be more apparent on qualitative reads for individual patients (66). ^18^F-FDG PET has an established role in the diagnosis of FTD, demonstrating frontal or anterior temporal-predominant hypometabolism (with sparing of the posterior cortical regions) in behavioral or language variants of FTD (66). In a head-to-head study of amyloid versus ^18^F-FDG PET in over 100 autopsy-confirmed cases (primarily AD and FTD), amyloid PET had higher sensitivity than ^18^F-FDG PET for the presence of AD neuropathology with similar specificity, although both modalities performed similarly in determining the causative neuropathology (67). ^18^F-FDG PET can also be useful in evaluating dementia with Lewy bodies (DLB) with occipital hypometabolism and preserved metabolism in the posterior cingulate (“cingulate island sign”), helping to distinguish the metabolic pattern from that of AD (68–70). Characteristic patterns have also been reported in atypical parkinsonian syndromes, such as CBD, PSP, and multiple system atrophy (71).

Presynaptic dopaminergic imaging (e.g., ^123^I-DaTscan single photon emission tomography [SPECT] or ^18^F-FDOPA-PET) supports the differential diagnosis between DLB and AD by demonstrating loss of dopaminergic cells in the nigrostriatal pathway, with decreased radiotracer uptake in the putamen and caudate. There is ∼80% sensitivity and ∼92% specificity for the diagnosis of DLB compared with neuropathological diagnoses obtained at autopsy (72*,*^73^). However, presynaptic dopaminergic denervation can be present in neurodegenerative causes of parkinsonism other than DLB.

Apart from the most commonly used PET tracers, other PET tracers are being developed with high potential in dementia research. These include markers of neuroinflammation (74*,*^75^) and synaptic density (76). PET radiotracers that bind to other protein aggregates associated with neurodegeneration, such as α-synuclein and TAR DNA-binding protein 43 (TDP-43), are currently in early stages of development (77–79).

### 6.2 Fluid Biomarkers of Amyloid and Tau

Different isoforms of amyloid can be reliably measured in CSF, where the levels of Aβ42 are reduced by 40%–60% in individuals with amyloid plaques compared with the levels in amyloid-negative controls, whereas CSF Aβ40 levels do not discriminate patients with and without plaque deposition (80). CSF measures of total tau and phosphorylated tau (P-tau; at residues 181 or 217) levels are elevated in patients with AD. Elevated total tau levels are not specific to AD and are also seen in other conditions associated with neuronal injury, including stroke, traumatic brain injury, and Creutzfeldt-Jakob disease. Elevated CSF P-tau181 and P-tau217 levels are more specific for AD and may reflect amyloid-mediated changes in tau phosphorylation and secretion (81*,*^82^).

Numerous studies have shown a high concordance between amyloid PET imaging and CSF Aβ42/Aβ40 and Aβ42/P-tau181 ratios (see for example (83*,*^84^)). These CSF ratios perform better than concentrations of Aβ42 or P-tau alone for predicting amyloid PET status (84*,*^85^). Across the AD continuum, CSF P-tau, especially P-tau217, is moderately associated with the load of both amyloid and tau PET (86*,*^87^). Alternative tau assays, such as P-tau205 and (in particular) microtubule-binding region of tau at residue 243 (MTBR-tau243), may track better with NFT deposition and tau PET (88), but are not yet available outside of research studies.

When the clinically approved high-precision CSF assays are used, the CSF Aβ42/Aβ40 (or Aβ42/p-tau) ratio can predict the visual classification of amyloid PET images with similar accuracy to quantitative assessments (SUVRs) of the same PET images (84). Not surprisingly, amyloid PET and CSF AD ratios detect early AD with similar accuracy, and there is no added value to combining the 2 measures to detect amyloid positivity (89). Fully automated CSF AD biomarker assays have recently been approved by the FDA and other regulatory authorities.

In recent years, major advances have been made in developing high-precision plasma assays for AD biomarkers (90). Mass spectrometry-based methods for quantification of Aβ42/Aβ40 in plasma have shown high correlation with CSF amyloid biomarkers or amyloid PET (91*,*^92^). However, the levels of plasma Aβ42/Aβ40 are decreased by only 8%–15% in individuals with cerebral amyloid pathology versus the 40%–60% decreases seen in CSF. Therefore, the robustness of plasma Aβ42/Aβ40 at the individual patient level may be suboptimal for clinical use (93*,*^94^). In contrast, plasma P-tau levels (measured by high-sensitivity immunoassays) are increased by 3–7 times in cognitively impaired individuals with AD compared with levels in CU controls (90). Measurement of plasma tau phosphorylated at various epitopes, including P-tau181, P-tau217, and P-tau231, has high accuracy in differentiating cognitive impairment due to AD from cognitive impairment caused by other conditions, with plasma P-tau217 consistently showing the highest diagnostic performance (95–102). Further, plasma P-tau217 can be used to predict future development of AD dementia in nondemented symptomatic (103*,*^104^) and CU individuals (105*,*^106^). Several studies have also shown that plasma P-tau217 levels are highly concordant with amyloid PET positivity in both cognitively impaired (97*,*^102^*,*^107^) and cognitively unimpaired individuals (97*,*^108^–^110^). The use of mass spectrometry to measure the P-tau217 to non-P-tau ratio (%P-tau217) can detect both amyloid PET and tau PET positivity with areas under the receiver operating characteristic curve of > 0.95. Further studies are needed to study how common medical comorbidities, such as kidney dysfunction or high body mass index, affect plasma AD biomarker levels in different populations (111). Current efforts are also underway to optimize plasma MTBR-tau243 as a fluid analog of tau PET (112).

Although biofluid and PET measures of amyloid and tau can both be useful for diagnostic purposes, it is important to note that CSF and plasma measurements reflect the concentrations of soluble forms of Aβ42 and P-tau, whereas PET radiotracers bind to aggregated protein inclusions. Several studies suggest that changes in CSF, plasma amyloid, and P-tau may be detectable earlier than PET changes (113*,*^114^). Although blood-based measures of amyloid, tau, and neurodegeneration are promising, they are not yet approved by the FDA for clinical use. For a comprehensive discussion on the current state of amyloid, P-tau, and other blood-based biomarkers of neurodegeneration (e.g., neurofilament light chain, glial fibrillary acidic protein, and others), see published AURs (13).

## 7. METHODS

### 7.1 Composition of Expert Workgroup

In June 2020, the AA and SNMMI convened a workgroup to update the AUC, with Avalere Health providing technical and editorial assistance. The workgroup participated in teleconference meetings on a biweekly basis through August 2021. An additional 1-time meeting was convened in August 2023 (see Section 7.5: Revisiting Clinical Scenarios Involving AD Therapeutics).

In alignment with the Institute of Medicine’s recommendations on group composition from its report *Clinical Practice Guidelines We Can Trust*, the AA and SNMMI established this multidisciplinary workgroup by including clinicians and other healthcare professionals with relevant expertise (115). The 14 members of the workgroup included 5 neurologists (GDR, DK, OH, SS, PH), 5 radiology/nuclear medicine physicians (JA, TB, KD, PHK, SM), 1 who was double-boarded in neurology and nuclear medicine (KJ), 1 PET imaging methodologist (JCP), 1 neuro-ethicist (JHL), and 1 pathology and laboratory medicine biomarker researcher (MEM). Twelve of the members were from the U.S. and 2 were from Europe (Spain and Sweden). Each member has published extensively on topics related to the key considerations around the use of amyloid and tau PET, such as dementia research, clinical practice and ethics, and biomarker test validation and clinical utilization. The complete list of workgroup members and disclosures of conflicts of interest accompany this publication, and the list of external reviewers in Appendix A.

### 7.2 Defining Scope and Key Research Questions

The process began with the workgroup defining the scope and parameters of the AUC and developing key research questions to guide a systematic review of available evidence on amyloid and tau PET using the PICOTS approach (population, interventions, comparisons, outcomes, timing, and settings framework) (116) (Appendix B).

The workgroup then developed a list of 17 clinical scenarios that are encountered in clinical practice based on key patient groups in whom amyloid or tau PET may be considered as part of the diagnostic process. The workgroup developed the clinical scenarios (Tables 2 and 3) through a confidential and formalized process adapted from the RAND and University of California, Los Angeles, approach for AUC development (117). The workgroup began by reviewing the clinical scenarios in the 2013 amyloid PET AUC (11), and then refining and updating the previous scenarios and adding several new ones. This resulted in an updated set of scenarios applicable for the consideration of amyloid and tau PET presented in this document.

**TABLE 2.** Clinical Scenarios and Appropriateness Ratings for Amyloid and Tau PET Imaging

	Rating*	
Clinical scenario	Amyloid PET	Tau PET
Clinical Scenario 1: Patients who are CU who are not considered to be at increased risk for AD based on age, known *APOE4* genotype, or multigenerational family history	1	1
Clinical Scenario 2: Patients who are CU but considered to be at increased risk for AD based on age, known *APOE4* genotype, or multigenerational family history	2	1
Clinical Scenario 3: Patients with SCD (cognitively unimpaired based on objective testing) who are *not* considered to be at increased risk for AD based on age, known *APOE4* genotype, or multigenerational family history	2	1
Clinical Scenario 4: Patients with subjective cognitive decline (CU based on objective testing) who are considered to be at increased risk for AD based on age, known *APOE4* genotype, or multigenerational family history	6	2
Clinical Scenario 5: Patients presenting with MCI or dementia syndrome who are younger than 65 y and in whom AD pathology is suspected	9	8
Clinical Scenario 6: Patients presenting with MCI or dementia syndrome that is often consistent with AD pathology (amnestic presentation) with onset at 65 y or older	8	6
Clinical Scenario 7: Patients presenting with MCI or dementia syndrome that could be consistent with AD pathology but has atypical features (e.g., nonamnestic clinical presentation, rapid or slow progression, etiologically mixed presentation)	8	7
Clinical Scenario 8: To determine disease severity or track disease progression in patients with an established biomarker-supported diagnosis of MCI or dementia due to AD pathology	1	4
Clinical Scenario 9: Patients presenting with prodromal Lewy body disease or DLB	2	4
Clinical Scenario 10: Patients with MCI or dementia with recent CSF biomarker results that are conclusive (whether consistent or not consistent with underlying AD pathology)	3	6
Clinical Scenario 11: Patients with MCI or dementia with equivocal or inconclusive results on recent CSF biomarkers	8	6
Clinical Scenario 12: To inform the prognosis of patients presenting with MCI due to clinically suspected AD pathology	8	7
Clinical Scenario 13: To inform the prognosis of patients presenting with dementia due to clinically suspected AD pathology	4	7
Clinical Scenario 14: To determine eligibility for treatment with an approved amyloid-targeting therapy	9^†^	8^†^
Clinical Scenario 15: To monitor response among patients who have received an approved amyloid-targeting therapy	8^†^	5
Clinical Scenario 16: Nonmedical usage (e.g., legal, insurance coverage, or employment screening)	1	1
Clinical Scenario 17: In lieu of genotyping for suspected autosomal dominant mutation carriers	1	1

* A score of 1–3 is rarely appropriate, of 4–6 is uncertain, and of 7–9 is appropriate.

† Scores reflect revoting in August 2023. See text for more details.

**TABLE 3.** Clinical Scenarios for Amyloid and Tau PET

Clinical scenarios for amyloid PET	Rating*
**Appropriate**	
Clinical Scenario 5: Patients presenting with MCI or dementia who are younger than 65 y and in whom AD pathology is suspected	9
Clinical Scenario 6: Patients presenting with MCI or dementia syndrome that is often consistent with AD pathology (amnestic presentation) with onset at 65 y or older	8
Clinical Scenario 7: Patients presenting with MCI or dementia syndrome that could be consistent with AD pathology but has atypical features (e.g., nonamnestic clinical presentation, rapid or slow progression, etiologically mixed presentation)	8
Clinical Scenario 11: Patients with MCI or dementia with equivocal or inconclusive results on recent CSF biomarkers	8
Clinical Scenario 12: To inform the prognosis of patients presenting with MCI due to clinically suspected AD pathology	8
Clinical Scenario 14: To determine eligibility for treatment with an approved amyloid-targeting therapy	9^†^
Clinical Scenario 15: To monitor response among patients who have received an approved amyloid-targeting therapy	8^†^
**Uncertain**	
Clinical Scenario 4: Patients with SCD (CU based on objective testing) who are considered to be at increased risk for AD based on age, known *APOE4* genotype, or multigenerational family history	6
Clinical Scenario 13: To inform the prognosis of patients presenting with dementia due to clinically suspected AD pathology	4
**Rarely Appropriate**	
Clinical Scenario 1: Patients who are CU who are not considered to be at increased risk for AD based on age, known *APOE4* genotype, or multigenerational family history	1
Clinical Scenario 2: Patients who are CU but considered to be at increased risk for AD based on age, known *APOE4* genotype, or multigenerational family history	2
Clinical Scenario 3: Patients with SCD (CU based on objective testing) who are *not* considered to be at increased risk for AD based on age, known *APOE4* genotype, or multigenerational family history	2
Clinical Scenario 8: To determine disease severity or track disease progression in patients with an established biomarker-supported diagnosis of MCI or dementia due to AD pathology	1
Clinical Scenario 9: Patients presenting with prodromal Lewy body disease or DLB	2
Clinical Scenario 10: Patients with MCI or dementia with recent CSF biomarker results that are conclusive (whether consistent or not consistent with underlying AD pathology)	3
Clinical Scenario 16: Nonmedical usage (e.g., legal, insurance coverage, or employment screening)	1
Clinical Scenario 17: In lieu of genotyping for suspected autosomal dominant mutation carriers	1

Clinical scenarios for tau PET	Rating*
**Appropriate**	
Clinical Scenario 5: Patients presenting with MCI or dementia who are younger than 65 y and in whom AD pathology is suspected	8
Clinical Scenario 7: Patients presenting with MCI or dementia syndrome that could be consistent with AD pathology but has atypical features (e.g., nonamnestic clinical presentation, rapid or slow progression, etiologically mixed presentation)	7
Clinical Scenario 12: To inform the prognosis of patients presenting with MCI due to clinically suspected AD pathology	7
Clinical Scenario 13: To inform the prognosis of patients presenting with dementia due to clinically suspected AD pathology	7
Clinical Scenario 14: To determine eligibility for treatment with an approved amyloid-targeting therapy	8^†^
**Uncertain**	
Clinical Scenario 6: Patients presenting with MCI or dementia syndrome that is often consistent with AD pathology (amnestic presentation) with onset at 65 y or older	6
Clinical Scenario 8: To determine disease severity or track disease progression in patients with an established biomarker-supported diagnosis of MCI or dementia due to AD pathology	4
Clinical Scenario 9: Patients presenting with prodromal Lewy body disease or DLB	4
Clinical Scenario 10: Patients with MCI or dementia with recent CSF biomarker results that are conclusive (whether consistent or not consistent with underlying AD pathology)	6
Clinical Scenario 11: Patients with MCI or dementia with equivocal or inconclusive results on recent CSF biomarkers	6
Clinical Scenario 15: To monitor response among patients who have received an approved amyloid-targeting therapy	5
**Rarely Appropriate**	
Clinical Scenario 1: Patients who are CU who are not considered to be at increased risk for AD based on age, known *APOE4* genotype, or multigenerational family history	1
Clinical Scenario 2: Patients who are CU but considered to be at increased risk for AD based on age, known *APOE4* genotype, or multigenerational family history	1
Clinical Scenario 3: Patients with SCD (CU based on objective testing) who are *not* considered to be at increased risk for AD based on age, known *APOE4* genotype, or multigenerational family history	1
Clinical Scenario 4: Patients with SCD (CU based on objective testing) who are considered to be at increased risk for AD based on age, known *APOE4* genotype, or multigenerational family history	2
Clinical Scenario 16: Nonmedical usage (e.g., legal, insurance coverage, or employment screening)	1
Clinical Scenario 17: In lieu of genotyping for suspected autosomal dominant mutation carriers	1

* A score of 1–3 is rarely appropriate, of 4–6 is uncertain, and of 7–9 is appropriate.

† Scores reflect revoting in August 2023. See text for more details.

### 7.3 Systematic Evidence Review Approach and Findings

In a parallel effort, the Pacific Northwest Evidence-based Practice Center at Oregon Health & Science University (OHSU) conducted a systematic review of the literature. The primary purpose of the review was to summarize and assess the strength of evidence for the safety, diagnostic accuracy, and effect on patient outcomes of amyloid and tau PET in cases posed in the key research questions listed in Appendix B.

Searches for the review were conducted by using Ovid MEDLINE without revisions (December 2020) and supplemented with a review of reference lists of relevant articles and systematic reviews. Database searches resulted in 3,238 potentially relevant articles. After a dual review of the abstracts and titles, 118 articles were selected for full-text dual review, and 18 studies (in 27 publications) were determined to meet inclusion criteria and were included in this review (Appendix C).

Two OHSU Evidence-based Practice Center staff reviewers independently assessed the quality of each study for inclusion. The strength of overall evidence was graded as high, moderate, low, or very low by using the GRADE method (Grading of Recommendations, Assessment, Development, and Evaluations), based on the quality of evidence, consistency, directness, precision, and reporting bias. Specifically, we adapted criteria from the U.S. Preventive Services Task Force for randomized trials and cohort studies and from the Quality Assessment of Diagnostic Accuracy Studies (118) for studies of diagnostic accuracy (Appendix D). Discrepancies were resolved through a consensus process.

### 7.4 Rating of Clinical Scenarios

Using the evidence summary, their clinical experience and expertise, and their knowledge of research outside of the scope of the evidence review, the workgroup used a modified Delphi approach to reach consensus on ratings for each of the clinical scenarios. This approach consisted of an online survey and 2 rounds of virtual scoring. When rating each scenario, workgroup members were asked to assess the benefits and risks to patients of using amyloid and tau PET imaging for the diagnosis of AD. In each scoring round, members were asked to assign to each clinical scenario a rating within ranges of appropriate, uncertain, or rarely appropriate for use of amyloid or tau imaging. A rating scale of 1 to 9 was used in each of the scoring rounds. The rating scale was defined as follows:

Score of 7 to 9, Appropriate:

9 - High confidence that use of the tracer is appropriate.

8 - Moderately confident that use of the tracer is appropriate.

7 - Only somewhat confident that the use of the tracer is appropriate.

Score of 4 to 6, Uncertain:

6 - Uncertain, but possibility that the use of the tracer is appropriate.

5 - Uncertain, evidence is inconclusive or lacking.

4 - Uncertain, but possible that the use of the tracer is rarely appropriate.

Score of 1 to 3, Rarely Appropriate:

3 - Only somewhat confident that the use of the tracer is rarely appropriate.

2 - Moderately confident that the use of the tracer is rarely appropriate.

1 - Highly confident that the use of the tracer is rarely appropriate.

After each round of voting, the resulting ratings given for each indication were tabulated and reported to the workgroup. When an indication received all 14 workgroup members’ ratings in a single category of Appropriate, Uncertain, or Rarely Appropriate, that indication was considered to have reached a consensus rating and was removed from the next round of voting. When voting for an indication resulted in all but 1 vote falling into the same category, that vote was considered an outlier and removed from the ratings.

The first round of voting was an anonymous online survey in which each member was asked to assign a single rating to each indication and enter a rationale for that rating. Workgroup members were then brought together for a series of 5 virtual meetings to complete the Delphi process. The virtual meetings began with a presentation of the first-round survey rating results and rationales. After extensive discussion, a second round of online voting was collected and tabulated. The results were reported to the workgroup for further discussion. In this final round of deliberation, the workgroup reached consensus on each indication, with all members rating the remaining indications as falling within the same category of Appropriate, Uncertain, or Rarely Appropriate.

### 7.5 Revisiting Clinical Scenarios Involving AD Therapeutics

Significant advances in AD therapeutics occurred following the initial round of scenario scoring and prior to publication of these updated AUC. These advances include the publication of positive pivotal phase 3 clinical trials of the anti-amyloid monoclonal antibodies lecanemab (15) and donanemab (16) and traditional FDA approval of lecanemab in July 2023 and donanemab in July 2024. Given the prominent role of amyloid PET (and to a lesser degree tau PET) in the clinical trials and future implementation of these therapies in clinical practice, the workgroup reconvened in August 2023 to revote on Clinical Scenarios 14 and 15, which pertain to the appropriateness of amyloid and tau PET to evaluate eligibility for, or monitoring response to, anti-amyloid therapeutics. Changes in scenario rankings between August 2021 and August 2023 are described in the text.

## 8. AUC FOR AMYLOID AND TAU PET CLINICAL SCENARIOS

### 8.1 Criteria for Clinical Scenarios

The following general principles served as the “litmus test” for appropriateness of amyloid or tau imaging across all clinical scenarios:

1. AD is considered a likely etiology of cognitive impairment, but the etiology remains uncertain after a comprehensive evaluation by a dementia expert.
2. Knowledge of the presence or absence of amyloid tau pathology is expected to help establish the etiology of impairment and alter management.

The workgroup recommends that these principles be met in all patients referred for clinical amyloid/tau PET across all clinical scenarios.

### 8.2 Anticipated Impact on Patient Care

The guiding principle for clinicians considering amyloid and tau PET is that the results of these studies should have a direct impact on patient care by aiding diagnosis of the cause of cognitive decline and thus guide patient management. Establishing the cause of impairment can inform the care plan in a variety of ways, including the following:

1. Determining eligibility for drug treatment (e.g., approved and emerging molecular-specific therapies for AD and approved AD symptomatic treatments that are not indicated in other disorders).
2. Counseling the patient and family regarding prognosis.
3. Reducing the need for alternative diagnostic tests for AD (e.g., CSF biomarkers) or initiating a workup for non-AD conditions.
4. Helping inform decisions about patient safety (e.g., independent living, driving) and future planning (e.g., initiating or activating advance directives).

The workgroup strongly emphasized the “value of knowing” in patients seeking care for cognitive changes (119–121), beyond concrete changes in patient management. Furthermore, amyloid and tau PET results can determine whether a patient is eligible to participate in clinical research studies, including clinical trials.

In evaluating the utility of amyloid and tau PET, clinicians should consider patient-specific factors such as stage of impairment and age. Generally speaking, determining amyloid and tau status is more useful in the early stages of impairment and may be less impactful in patients who already have moderate-to-severe dementia. Although tau PET positivity is more strongly linked to cognitive symptoms, the prevalence of amyloid PET positivity increases with age in CU people, ranging in prevalence from ∼10% at age 50 to ∼45% at age 90 (122*,*^123^). In each age strata, the likelihood of amyloid PET positivity is 2–3 times higher in individuals who carry 1 or more copies of the apolipoprotein E ε4 risk allele (*APOE4*) than in *APOE4* non-carriers. Therefore, whereas a *negative* amyloid PET scan is always useful for ruling out AD, the clinical relevance of a positive scan should take into account a patient’s cognitive status, age, and the baseline prevalence of amyloid positivity in similarly aged unimpaired individuals.

The decision to pursue amyloid or tau PET should result from shared decision making between the ordering clinician, patient, and family and should take into account the patient’s and family’s desire to know the amyloid/tau status in light of each possible test outcome (including positive, negative, or indeterminate results). Although current data, obtained primarily in research settings, suggest that amyloid PET results can be disclosed safely and do not typically cause psychological harm, the individual mental health circumstances and support networks of the imaging candidate should be considered. Finally, as insurance coverage for amyloid and tau PET remains uncertain for many patients, the decision-making process should address the potential for co-payment and other out-of-pocket costs (124*,*^125^).

Although the workgroup sought to highlight the most common clinical scenarios under which amyloid and tau PET may be considered, a limited number of standardized scenarios can never capture the heterogeneity of patients in clinical practice, nor convey the complexity of clinical decision making for individual patients. Therefore, the criteria presented here should be considered as guidelines for clinicians, but not as a substitute for careful clinician judgment that considers the full clinical context for each patient who presents with cognitive complaints. In developing the scenarios, the workgroup considered the degree to which PET results would inform patient diagnosis and care from the available literature most relevant to the scenario’s clinical circumstance.

### 8.3 Clinical Scenarios and Appropriateness Ratings for Amyloid and Tau PET Imaging

The appropriateness scores (based on majority vote on the appropriateness scale at the conclusion of the Delphi process) for each clinical scenario are presented in Table 2. The overall categorizations of each scenario as appropriate, uncertain, or rarely appropriate for each modality are presented in Table 3. *It is important to note that each of the ratings for the clinical scenarios presented below reflect the level of appropriate use of each modality by itself: amyloid imaging independent or in the absence of tau imaging, and tau imaging independent or in the absence of amyloid imaging.* The use of both modalities in combination is discussed later in the document (see Section 9: Value of Tau PET Imaging in Combination with Amyloid PET Imaging). In addition, although several studies have evaluated the clinical impact of amyloid PET, there is a paucity of data about clinical uses of tau PET, which to date has primarily been used in research studies. As a result, workgroup recommendations regarding tau PET were often based on expert opinion and are not yet supported by empirical evidence. Therefore, the workgroup generally had lower confidence in the appropriateness of tau PET in most scenarios.

### 8.4 Rationale for Clinical Scenario Appropriateness Ratings

**Clinical Scenario 1**

**Patients who are CU, who are not considered to be at increased risk for AD based on age, known APOE4 genotype, or multigenerational family history**

Consensus ratings

Amyloid - 1 Highly confident that the use of the tracer is rarely appropriate.

Tau - 1 Highly confident that the use of the tracer is rarely appropriate.

**Amyloid**

This scenario refers to CU individuals (Section 3: Key Definitions) who are not at heightened risk of developing AD based on their age, *APOE* genotype, or family history. As discussed earlier, a significant minority of such individuals will have positive amyloid PET scans. This preclinical stage of AD is an area of active investigation in both observational research and drug trials aimed at the prevention of future cognitive decline. Group-level analyses clearly indicate that amyloid PET-positive CU individuals show accelerated cognitive decline compared with amyloid PET-negative CU individuals and are at heightened risk of developing MCI or dementia (126–128) (see Section 11: Further Research Questions). However, at the individual patient level, there remains significant uncertainty about cognitive outcomes, and many amyloid-positive individuals do not develop clinically meaningful cognitive impairment even with relatively extended follow-up (129). Currently, the uncertain clinical utility outweighs any benefits, although the availability of proven preventive therapies would undoubtedly alter this judgment. Consequently, the workgroup classified this indication as rarely appropriate (rating = 1).

**Tau**

The vast majority of CU individuals will show either completely negative tau PET results or retention limited to the medial temporal lobe but sparing the neocortex; this is insufficient for a positive tau PET read based on the FDA-approved visual read criteria (Figure 2) (130–133). Tau PET uptake outside the medial temporal lobe is exceedingly rare in individuals who have negative amyloid PET results. Emerging data suggest that individuals who have positive results for both amyloid *and* tau PET scans are at higher risk of imminent cognitive decline compared with patients who have positive results on just 1 of the 2 scans, or negative results on both (132*,*^133^). Up to 50% of amyloid-negative individuals show isolated tau PET uptake in the medial temporal lobe, and these individuals as a group show slower clinical decline compared with those with medial temporal tau *and* amyloid PET positivity (134). Clearly, there is much yet to learn in terms of how best to apply tau PET along the continuum of cognitive functioning, alone and in tandem with amyloid imaging. From the paucity of data, especially regarding individual patient risk, the workgroup classified tau PET as rarely appropriate in this scenario (rating = 1).

**Clinical Scenario 2**

**Patients who are CU but considered to be at increased risk for AD based on age, known APOE4 genotype, or multigenerational family history**

Consensus ratings

Amyloid - 2 Moderately confident that the use of the tracer is rarely appropriate.

Tau - 1 Highly confident that the use of the tracer is rarely appropriate.

**Amyloid**

Amyloid positivity is associated with age, family history, and *APOE4* genotype (123*,*^135^). Furthermore, age and *APOE4* genotype increase the risk of developing MCI or dementia in CU individuals who have positive results for amyloid PET (135–137). These individuals may be more likely to seek memory specialist care to determine their risk of developing AD because of family history or known genetic risk, as *APOE* testing is available through several straight-to-consumer genetic testing platforms. Current recommendations to ameliorate risk of cognitive decline due to AD or other causes involve optimizing treatment of vascular risk factors, in addition to lifestyle factors that highlight the importance of physical, cognitive, and social activity; diet; and adequate sleep. These recommendations are universal regardless of an individual’s risk of AD or amyloid status. As a result, the workgroup concluded that amyloid PET would be rarely appropriate in this scenario, acknowledging that this is an evolving clinical decision point affected by the need to know and by the possibility of future preventive pharmacological interventions (rating = 2).

**Tau**

As described in Scenario 1, currently available information about the utility of tau PET in this scenario is limited. The workgroup concluded that tau PET is rarely appropriate in this scenario (rating = 1).

**Clinical Scenario 3**

**Patients with SCD (CU based on objective testing) who are not considered to be at elevated risk for AD based on age, known APOE4 genotype, or multigenerational family history**

Consensus ratings

Amyloid - 2 Moderately confident that the use of the tracer is rarely appropriate.

Tau - 1 Highly confident that the use of the tracer is rarely appropriate.

**Amyloid**

Subjective cognitive decline (SCD) (Section 3: Key Definitions (138)) is common (139). In general, having SCD doubles the risk of developing MCI (140*,*^141^), but the time lag from detection of SCD to MCI averaged 9.4 years (SD 12.1 years) in 1 study (142). In one cohort, incident MCI occurred in only 4 of 318 (1%) SCD participants after 24 months (142). Persons with SCD who seek evaluation in a memory clinic may be at higher risk of decline than are individuals with SCD in the general population (143). The clinically defined construct of SCD covers a surprisingly wide spectrum of phenomena that could be construed as representing a change from prior level of function. Some (140), but not all, studies show that carriage of an *APOE4* allele increases the risk of decline. Higher age, especially over age 80 years, is predictive of greater risk. On clinical grounds, the greater the consistency and breadth of cognitive complaints, the higher the likelihood of subsequent development of MCI (141). Because of the long delay between detection of SCD and objective cognitive impairment, the highly variable likelihood of developing it, and the frequent presence of amyloid in an otherwise “normal” population, biomarker evidence of risk in SCD is necessarily of less certain prognostic value. Prognostic value of imaging biomarkers for AD in SCD is a complex function of length of time horizon, age, and presence of comorbidities.

Elevated amyloid is at least as common among persons > 65-years-old with SCD as in CU persons and may be slightly (but not dramatically) higher (144–147), is probably an interaction between the magnitude of SCD and amyloid burden (148*,*^149^), and might predict more cognitive impairment (150). The workgroup members, in noting that elevated amyloid conveyed little prognostic information and no actionable preventive interventions in persons with SCD who lacked an *APOE4* allele or multigenerational family history, felt that amyloid imaging is rarely appropriate (rating = 2).

**Tau**

Because elevations in tau PET are so closely tied to the degree of cognitive impairment, the probability of meaningfully elevated tau PET (outside of the medial temporal lobe) is very low in persons with SCD (145), who by definition have normal objectively measured cognition. Therefore, tau PET was considered by the workgroup to be rarely appropriate (rating = 1).

**Clinical Scenario 4**

**Patients with SCD (CU based on objective testing) who are considered to be at increased risk for AD based on age, known APOE4 genotype, or multigenerational family history**

Consensus ratings

Amyloid - 6 Uncertain, but possibility that the use of the tracer is appropriate.

Tau - 2 Moderately confident that the use of the tracer is rarely appropriate.

**Amyloid**

As discussed in Scenario 3, persons with SCD who are older, carry the *APOE*e*4* risk allele, or have a multigenerational family history are at higher risk of developing MCI/dementia. In these individuals, SCD is more likely to represent the earliest symptomatic stages of AD. Both positive and negative amyloid PET results may be informative to these individuals. Nevertheless, because the degree of individual risk and the time course for developing impairment are highly uncertain (89*,*^126^*,*^136^) in this population, preventive measures are limited to generally applicable lifestyle and health recommendations. Balancing these competing factors, the workgroup was ultimately uncertain but endorsed the possibility that amyloid PET may be appropriate in this scenario (rating = 6).

**Tau**

Even in persons with risk factors such as older age, *APOE4* genotype, or multigenerational family history, the probability of meaningfully elevated tau outside of the medial temporal lobe is very low in persons with SCD (145), who by definition have normal objectively measured cognition. Therefore, tau PET was considered by the workgroup to be rarely appropriate (rating = 2).

**Clinical Scenario 5**

**Patients presenting with MCI or dementia who are younger than 65 years and in whom AD pathology is suspected**

Consensus ratings

Amyloid - 9 High confidence that use of the tracer is appropriate.

Tau - 8 Moderately confident that use of the tracer is appropriate.

**Amyloid**

Young-onset dementia or MCI is defined as individuals who present with cognitive impairment before the age of 65 (151). A recent meta-analysis identified the prevalence of young-onset dementia in ages 30–64 to be 119.0 per 100,000 persons, with AD being the leading cause, followed by FTD and vascular dementia (152). Although the age cutoff of 65 is arbitrary, neuropathological evidence suggests greater amyloid and tau burden in younger than in older individuals affected by AD (153*,*^154^). As these working-aged individuals are in the prime of life and are often supporting families, accurately diagnosing the cause of impairment is particularly important. The greater frequency of atypical (non-amnestic) clinical presentations in young-onset AD (24), involving initial impairment in executive, language, visual, and (more rarely) behavior or motor function, often leads to delays in diagnosis or misdiagnosis that affects treatment (155*,*^156^). Given the lower frequency of coexisting pathologies in young-onset AD brains (157), this population may be more likely to benefit from specific therapeutic agents targeting amyloid and tau.

Amyloid PET is highly accurate in detecting AD neuropathology in patients with young-onset impairment. Rates of amyloid positivity are much lower in this age group in CU people or patients with other neurodegenerative syndromes (67*,*^123^*,*^158^). Conversely, in patients presenting clinically with an amnestic dementia, the prevalence of amyloid PET positivity *decreases* with increasing age due to a higher prevalence of non-AD neuropathologies that affect the medial temporal lobe (e.g., limbic-predominant age-related TDP-43 encephalopathy [LATE]) (123*,*^159^). Taken together, in the setting of a clinical syndrome suggestive of AD, amyloid PET positivity in young-onset dementia and MCI can be helpful for ruling in AD as the underlying neuropathology. Overall, the workgroup concluded that amyloid PET is appropriate in this scenario (rating = 9).

**Tau**

Similarly, tau PET can be helpful in detecting AD pathology in young-onset AD, with higher overall intensity and spatial spread of radiotracer retention compared with that in older patients at a similar disease stage (160). Patients with young-onset AD are more likely to be in advanced Braak stages of neurofibrillary pathology even at the MCI stage (160), increasing the likelihood of a positive tau PET scan (10*,*^161^*,*^162^). Furthermore, variability in tau PET retention patterns closely mirrors the variability seen in neurodegeneration patterns (via MRI or ^18^F-FDG PET) in young-onset AD (158*,*^163^*,*^164^). Overall, from the current evidence, the workgroup concluded that tau PET is appropriate in this scenario (rating = 8).

**Clinical Scenario 6**

**Patients presenting with MCI or dementia syndrome that is often consistent with AD pathology (amnestic presentation) with onset at 65 years or older**

Consensus ratings

Amyloid - 8 Moderately confident that use of the tracer is appropriate.

Tau - 6 Uncertain, but possibility that the use of the tracer is appropriate.

**Amyloid**

This scenario addresses cognitively impaired older adults who meet clinical criteria for MCI or a dementia syndrome that is amnestic in presentation and otherwise consistent with AD. In the original amyloid PET AUC, it was felt that amyloid PET would not add much value in individuals with dementia who have symptoms and an age of onset that is typical of AD (11). However, subsequent reports from both observational studies and drug trials reported that 15%–20% of individuals clinically diagnosed with late-onset probable AD dementia (including ∼35% of *APOE4*-negative individuals) have negative amyloid PET results (165*,*^166^). Interestingly, the prevalence of amyloid PET positivity *decreases* with older age in patients with clinically typical amnestic dementia, likely reflecting an increasing prevalence of non-AD pathologies (e.g., vascular, LATE) that can mimic AD clinically (123). The rates of amyloid PET positivity in late-onset MCI range from 45% to 70% (167), increasing with age and *APOE4* genotype. Thus, there is almost always diagnostic uncertainty about the contribution of AD at the MCI stage. As discussed earlier, amyloid positivity is also common in CU older adults and may be less specific among older patients in general. With advanced age comes an increasing likelihood that medical comorbidities and/or other coexisting pathologies (including overlapping neurodegenerative diseases) are contributing to the clinical presentation of cognitive impairment (25). Nevertheless, a positive scan can, by virtue of satisfying the biomarker criteria required for a diagnosis of AD in persons with MCI or dementia, reduce the need for further diagnostic testing and heighten confidence in the management approach. In contrast, a negative scan can serve to rule out AD pathology as a cause of the observed impairment, triggering an alternative course for the diagnostic workup and resulting management plan. In the Imaging Dementia-Evidence for Amyloid Scanning (IDEAS) study, amyloid PET imaging was positive in 55.3% of patients with MCI over age 65 and led to changes in patient management in 60.2% of these patients (165). From these data, the workgroup concluded that amyloid PET is appropriate in this scenario (rating = 8).

**Tau**

The workgroup acknowledged the mounting data supporting the accuracy of tau PET for identifying pathological changes of AD and the high predictive value (i.e., correlation with a histopathological reference standard) of such findings for patients presenting with dementia (10*,*^161^). However, given the evidence that a positive ^18^F-FTP tau PET result (as rated by FDA-approved visual read criteria) reliably detects primarily advanced stages of tau pathology (Braak stages V–VI), a negative FTP tau PET visual read does not exclude the presence of clinically meaningful tau pathology (i.e., Braak stages III–IV), which represents the median tau pathology seen at autopsy in patients who died with MCI, as well as in some patients who died with dementia (161). In contrast to that for amyloid PET, the *positive predictive value* of FTP tau PET in patients with MCI or dementia is high, whereas the *negative predictive value* is uncertain, especially in older patients who may develop impairment at lower levels of tau pathology. The workgroup also acknowledged the need for additional research on the utility of tau PET for clinical decision making in cognitively symptomatic patients at both the MCI and dementia stages of impairment. Ultimately, the workgroup was uncertain but endorsed the possibility that FTP tau PET may be appropriate in this scenario (rating = 6).

**Clinical Scenario 7**

**Patients presenting with MCI or dementia syndrome that could be consistent with AD pathology but has atypical features (e.g., non-amnestic clinical presentation, rapid or slow progression, etiologically mixed presentation)**

Consensus ratings

Amyloid - 8 Moderately confident that use of the tracer is appropriate.

Tau - 7 Only somewhat confident that the use of the tracer is appropriate.

**Amyloid**

Symptomatic cognitive impairment due to AD is clinically heterogenous. Although memory loss is the most common presenting symptom, an estimated 20%–25% of patients present with non-amnestic syndromes, including primary changes in language (168), visuospatial/visuoperceptual abilities (169), executive functioning (170), and (more rarely) changes in personality, behavior, and motor functioning (24*,*^171^–^173^). Autopsy studies suggest that AD is the most common underlying neuropathology in patients presenting with the logopenic variant of primary progressive aphasia (lvPPA) (174*,*^175^) and posterior cortical atrophy (PCA) syndromes (55*,*^176^). AD is also associated with a primary dysexecutive syndrome (170) and is the underlying neuropathology in ∼25% of patients presenting with corticobasal syndrome (CBS) (177). AD pathology is a relatively rare cause of the behavioral variant of FTD (173*,*^178^) and nonfluent/agrammatic or semantic variants of PPA (174*,*^175^). Furthermore, although AD is typically associated with a slow and insidious decline in cognition and function, some patients present with unusually rapid or slow progression (58*,*^179^). Finally, mixed pathologies are increasingly common in older patients with MCI and dementia (157*,*^180^), and these pathologies can manifest as clinically mixed presentations, with features of both AD and other dementia syndromes.

Patients presenting with atypical features often present a diagnostic challenge. Amyloid PET can be helpful in excluding AD neuropathology in these patients (67*,*^123^). A negative amyloid PET scan may increase clinical suspicion of a non-AD neurodegenerative process such as frontotemporal lobar degeneration (FTLD), particularly in patients presenting with focal non-amnestic syndromes (181). In patients with mild impairment and slow progression, a negative amyloid PET scan raises the possibility of a potentially treatable, nondegenerative cause of impairment (e.g., primary medical, mood, or sleep disorder). Conversely, in patients with rapid progression, a negative amyloid PET scan may suggest a non-AD neurodegenerative disease, prion disease, or autoimmune encephalopathy. A positive amyloid PET scan increases the likelihood that AD is the primary cause of impairment (particularly in lvPPA and PCA, in which the a priori likelihood of AD is high), or a contributing pathology in patients with etiologically mixed presentations. As always, the patient’s age should be considered in interpreting the clinical meaningfulness of a positive amyloid PET result, given the increasing prevalence of amyloid in CU individuals with increasing age (167). In the IDEAS study, 70.1% of patients with atypical dementia were positive for amyloid PET, leading to changes in management in 63.5% of these patients (165). Overall, the workgroup concluded that amyloid PET was appropriate in this scenario (rating = 8).

**Tau**

As with amyloid PET, an “AD-like” tau PET binding pattern can help establish AD as a primary or contributing cause of impairment (10*,*^161^*,*^162^). Furthermore, the spatial pattern of tau PET often matches brain regions that are clinically affected and show evidence of neurodegeneration on FDG PET or MRI (e.g., greater involvement of occipital visual processing regions in PCA, greater left hemisphere involvement in lvPPA, and greater binding in the sensorimotor cortex in CBS due to AD) (61*,*^182^–^184^), increasing confidence that the underlying syndrome is due to AD. In addition, a high tau burden is associated with more rapid clinical progression and a low tau burden with slower progression (185*,*^186^). ^18^F-FTP shows absent-to-low binding to tau aggregates in non-AD tauopathies (e.g., chronic traumatic encephalopathy or tau subtypes of FTLD) (162*,*^187^*,*^188^), but tau PET should not be used clinically to rule in these conditions. Overall, the workgroup concluded that tau PET was appropriate in this scenario (rating = 7).

**Clinical Scenario 8**

**To determine disease severity or track disease progression in patients with an established biomarker-supported diagnosis of MCI or dementia due to AD pathology**

Consensus ratings

Amyloid - 1 Highly confident that the use of the tracer is rarely appropriate.

Tau - 4 Uncertain, but possible that the use of the tracer is rarely appropriate.

**Amyloid**

This scenario relates to patients with an *existing* diagnosis of MCI or dementia due to AD pathology supported by biomarker evidence, for example, a positive amyloid PET scan or a CSF profile consistent with AD. Cross-sectional and longitudinal studies do not support the use of a subsequent amyloid PET to assess the degree of cognitive impairment or to monitor the rate of progression of the underlying AD pathological process. Both autopsy and PET studies have shown that amyloid accumulation begins approximately 2 decades before onset of cognitive decline (167), proceeds in a sigma-shaped fashion, is substantial at the MCI stage, and has typically approached a plateau at the stage of mild AD dementia (136*,*^189^). There is little further accumulation as clinical manifestations progress, and so serial scans are not helpful to monitor disease progression. In addition, since there is little correlation between the level of brain amyloid and cognitive function in MCI or AD (190), a repeat scan will not provide information on disease severity. Disease severity and progression in patients in this scenario should be tracked by clinical evaluation, including cognitive testing.

Because a subsequent amyloid scan provides no actionable information about disease severity or progression in patients with a biomarker-supported diagnosis of MCI or dementia due to AD pathology, the workgroup concluded that amyloid PET is rarely appropriate in this clinical scenario (rating = 1).

**Tau**

In contrast to that for amyloid PET, autopsy and PET studies have shown that the level of cortical tau correlates with cognitive status and symptomatic disease stage (1*,*^2^*,*^191^). However, data are limited on the clinical utility of serial tau scans. Therefore, the use of tau PET scans to track disease progression is uncertain. Currently, such a scan would not change patient management or add additional useful information beyond what is provided by serial clinical evaluations, for example, with cognitive testing. It is possible that changes in tau PET could inform prognosis or treatment choices, but this remains to be demonstrated. The method of scan interpretation may play a role in considering the potential utility of serial tau scans. Both quantitative approaches and visual assessment of progression in the spatial pattern of tau could be useful. In addition, it should be noted that serial tau scans can have great value as a clinical research tool or in anti-AD drug development, as they can reflect disease progression or response to therapy. Overall, from currently available data, the workgroup was uncertain but endorsed the possibility that tau PET may rarely be appropriate in this scenario (rating = 4).

**Clinical Scenario 9**

**Patients presenting with prodromal Lewy body disease or DLB**

Consensus ratings

Amyloid - 2 Moderately confident that the use of the tracer is rarely appropriate.

Tau - 4 Uncertain, but possible that the use of the tracer is rarely appropriate.

**Amyloid**

Dementia with Lewy bodies (DLB) is characterized by predominant deficits in executive and visuospatial functions, accompanied by additional core clinical features, including 1 or more spontaneous features of parkinsonism, fluctuating cognition, visual hallucinations, and rapid eye movement (REM) sleep behavior disorder (192). Biomarkers contributing to the diagnosis are (1) reduced binding of dopamine transporter radioligands in basal ganglia on SPECT or PET imaging, (2) low uptake of iodine-131 meta-iodobenzylguanidine on myocardial scintigraphy, and (3) polysomnographic confirmation of REM sleep without atonia. Novel CSF seed amplification assays may provide direct evidence for aggregation of α-synuclein, the protein deposited in Lewy bodies and Lewy neurites (193). The diagnosis of DLB is appropriate when dementia precedes or occurs concurrently with parkinsonism, whereas a diagnosis of Parkinson’s disease with dementia (PDD) is more appropriate when dementia occurs in the setting of established Parkinson’s disease (typically at least 1 year prior to dementia). Proposed criteria for prodromal MCI with LB (MCI-LB) include MCI (particularly involving executive or visuospatial domains with relative sparing of episodic memory) occurring in combination with core DLB clinical and biomarker features. Less well-characterized prodromal DLB presentations are delirium or marked fluctuations in consciousness and late-onset psychiatric presentations, including major depression or psychosis (194). The defining neuropathology of DLB is widespread limbic and neocortical α-synuclein-containing Lewy bodies and Lewy neurites. Approximately 50% of patients with DLB are found to have core features of AD neuropathology, including diffuse and neuritic amyloid plaques and tau NFTs. Given the high prevalence of co-pathology, AD-specific biomarkers such as amyloid and tau PET are in general not useful in the diagnostic evaluation of DLB.

Amyloid PET is positive in over 50% of patients with DLB (123), corresponding to the high prevalence of amyloid plaques (diffuse more than neuritic plaques) at autopsy. Previous studies reported rates of 35%–40% amyloid PET positivity in patients with MCI-LB (195). As in other disorders, amyloid positivity is more common with increased age and the presence of the *APOE4* genotype. The pattern of amyloid tracer uptake is similar to that of AD, whereas binding intensity is on average intermediate between controls and those with dementia due to AD (196). Overall, a positive amyloid PET scan does not help distinguish AD from DLB, although a negative scan can help exclude an AD diagnosis. Amyloid PET is more frequently positive in DLB than in PDD, and scan positivity is associated with lower cognitive performance and more rapid cognitive decline in PD, whereas results in DLB are mixed (196). Amyloid PET results may not influence drug treatment, since acetylcholinesterase inhibitors are indicated in both DLB and AD, and anti-amyloid antibody treatment would not be currently indicated in patients with clinical features of DLB. Overall, the workgroup concluded that amyloid PET is rarely appropriate in the evaluation of suspected DLB in its fully established or prodromal stages (rating = 2).

**Tau**

Tau NFT co-pathology is also often identified at autopsy in patients with PDD and DLB and contributes to cognitive impairment (197*,*^198^). The tau PET signal in DLB is on average intermediate between that in AD dementia and controls and higher than that in PDD (199–201). Tracer uptake is typically seen in the temporoparietal and occipital cortex, with relative sparing of the medial temporal lobes. Tau PET positivity is associated with amyloid PET positivity (although it is also seen in some amyloid-negative patients) and correlates with lower cognitive performance (202–205). A single small study of tau PET in prodromal DLB did not find elevated binding compared with that in controls (206). Overall, tau PET is unlikely to differentiate between DLB, PDD, and AD, although a positive scan increases the likelihood that AD pathology is contributing to cognitive impairment. As with amyloid PET, results of tau PET are unlikely to affect drug treatment. Overall, from a relatively small number of available studies, the workgroup was uncertain whether tau PET was appropriate in DLB, but felt it was possible that the indication was rarely appropriate (rating = 4).

**Clinical Scenario 10**

**Patients with MCI or dementia with recent CSF biomarker results that are conclusive (whether consistent or not consistent with underlying AD pathology)**

Consensus ratings

Amyloid - 3 Only somewhat confident that the use of the tracer is rarely appropriate.

Tau - 6 Uncertain, but possibility that the use of the tracer is appropriate.

**Amyloid**

When abnormal levels of brain amyloid are being determined, the CSF Aβ42/Aβ40 and P-tau181/Aβ42 ratios are highly congruent with the results obtained using amyloid PET imaging (207). Consequently, there is generally no need to perform an amyloid PET scan in patients with clearly abnormal or normal CSF biomarker ratios. However, amyloid PET does offer additional information beyond CSF biomarker ratios. Whereas CSF assays measure concentrations of soluble amyloid and P-tau monomers, amyloid PET characterizes the magnitude and spatial distribution of fibrillar amyloid plaque deposition. CSF may also detect amyloid-related changes prior to amyloid PET scan positivity. However, this additional information obtained from PET was felt to rarely lead to changes in diagnosis or management. Overall, the workgroup concluded that amyloid PET in this scenario is rarely appropriate (rating = 3). Although the group did not specifically discuss the utility of amyloid PET in patients with conclusive plasma AD biomarkers, similar principles would apply.

**Tau**

Few studies to date have evaluated the additional value of tau PET in patients with MCI and dementia with known CSF biomarker results. Even though CSF p-tau217 and p-tau181 concentrations correlate with the tau PET signal, the magnitude of correlation is modest; similar CSF concentrations can associate with highly variable degrees of tau PET uptake and spatial spread (86*,*^87^). In cognitively impaired patients, tau PET is more strongly associated with cognitive function than is CSF p-Tau concentration (81). Accumulating evidence indicates that CSF levels of p-tau change earlier than the tau PET signal in preclinical AD (208), reaching a relative plateau during the symptomatic stage of the disease (209*,*^210^), whereas the tau PET signal continues to increase in patients with AD dementia (211). Further, the fluid measures do not provide any regional information on tau pathology. Consequently, it is plausible that tau PET might add important information beyond CSF biomarkers, for example, for defining AD subtypes (212) and predicting subsequent cognitive decline (185), but additional studies are needed and the implications for patient care remain unclear. Overall, the workgroup was uncertain but endorsed the possibility that tau PET may be appropriate in this scenario (rating = 6). Although the group did not specifically discuss the utility of tau PET in patients with conclusive plasma AD biomarkers, similar principles would apply.

**Clinical Scenario 11**

**Patients with MCI or dementia with equivocal or inconclusive results on recent CSF biomarkers**

Consensus ratings

Amyloid - 8 Moderately confident that use of the tracer is appropriate.

Tau - 6 Uncertain, but possibility that the use of the tracer is appropriate.

**Amyloid**

Considering the bimodal distribution of the Aβ42/Aβ40 and P-tau/Aβ42 biomarker ratios, relatively few patients are close to the cutoffs used to define abnormality (83*,*^84^). However, in those patients with ratios very close to the established cutoffs, an amyloid PET scan could be considered to determine the Aβ status more confidently. The 2 ratios mentioned here are more accurate than single CSF biomarkers for determining brain amyloid status. For example, increased CSF P-tau levels in patients with clearly normal CSF Aβ42/Aβ40 and P-tau/Aβ42 ratios do not usually warrant an amyloid PET scan. Overall, the workgroup concluded that amyloid PET is appropriate in this scenario (rating = 8). Although the workgroup did not discuss the utility of amyloid PET in patients with equivocal or inconclusive plasma AD biomarkers, similar principles would apply.

**Tau**

In Scenario 10, it was concluded that tau PET might have additional value independent of the outcome of already obtained CSF biomarker results. The workgroup reached a similar conclusion for Scenario 11, expressing uncertainty but endorsing the possibility that tau PET may be appropriate in this scenario (rating = 6). Although the workgroup did not discuss the utility of tau PET in patients with equivocal or inconclusive plasma AD biomarkers, similar principles would apply.

**Clinical Scenario 12**

**To inform the prognosis of patients presenting with MCI due to clinically suspected AD pathology**

Consensus ratings

Amyloid - 8 Moderately confident that use of the tracer is appropriate.

Tau - 7 Only somewhat confident that the use of the tracer is appropriate.

**Amyloid**

There is robust evidence of the prognostic value of amyloid PET for predicting future outcomes in patients with MCI whose clinical presentation is amnestic or otherwise consistent with AD. Although definitions of MCI subtypes are variable across studies, numerous reports have found that, allowing for adequate follow-up duration, a majority of MCI patients with a positive amyloid PET scan will progress to AD dementia, whereas the risk of progression to AD dementia is significantly lower in those who are amyloid negative (213–219). Overall, a positive amyloid PET scan at baseline is associated with an average hazard ratio of ∼3–4 (range: 2.1–11.4) for conversion to dementia in studies with 1–4.5 years of follow-up, after adjusting for confounding variables. The value of amyloid PET for informing prognosis in MCI is further supported by studies documenting the marked uncertainty and, in some cases, emotional turmoil that persons with MCI and their family care partners live with daily (220). Learning whether or not AD pathology is present may lessen such uncertainty and enable clinicians and family care partners to guide patients with amyloid positivity to available resources for future planning. However, evidence is limited, and 1 study found that disclosure of amyloid PET results did not alter perceptions of ambiguity among patients and families affected by MCI (220). The workgroup acknowledged that the “value of knowing” one’s brain amyloid status in the context of MCI is a theoretical construct about which high-level empirical evidence is lacking. Furthermore, individual rates of clinical progression in patients with amyloid-positive MCI are highly variable (221), and the prognostic value of amyloid PET may be improved if combined with MRI or ^18^F-FDG PET as imaging markers of neurodegeneration (66). Although a positive amyloid PET scan is useful in predicting *whether* individuals are likely to progress to dementia, it is not as useful at predicting *time to conversion*, and individuals with a negative amyloid PET scan may still develop a non-AD dementia. Despite these caveats, the workgroup concluded that amyloid PET is appropriate in this scenario (rating = 8).

**Tau**

Cohort studies have consistently found a positive tau PET scan to be associated with an increased likelihood of cognitive and functional decline in persons with MCI, suggesting the potential for such testing to inform prognosis in this clinical scenario. In a recent large multisite study, tau PET was a stronger predictor of longitudinal cognitive decline than was amyloid PET or MRI cortical thickness in individuals with amyloid-positive MCI (185). However, the use of tau PET in this scenario is currently being prospectively validated, and additional longitudinal studies are needed to further elucidate the prognostic value of tau PET in MCI. Overall, the workgroup was somewhat confident that tau PET is appropriate in this scenario (rating = 7).

**Clinical Scenario 13**

**To inform the prognosis of patients presenting with dementia due to clinically suspected AD pathology**

Consensus ratings

Amyloid - 4 Uncertain, but possible that the use of the tracer is rarely appropriate.

Tau - 7 Only somewhat confident that the use of the tracer is appropriate.

**Amyloid**

The value of amyloid PET lies predominantly in confirming the presence of AD pathology as opposed to providing prognostic value. As a group, persons who meet clinical criteria for dementia due to AD and have a positive amyloid PET scan decline more rapidly than do those who meet clinical criteria but have a negative amyloid PET scan (222). This finding likely represents the fact that non-AD neuropathologies that mimic AD clinically (e.g., LATE) are associated with less rapid decline. However, in amyloid-positive individuals with dementia, amyloid deposition has often plateaued and the burden or distribution of amyloid correlates poorly with the baseline level of impairment or subsequent longitudinal decline (223). Overall, the workgroup was uncertain but endorsed the possibility that amyloid PET may rarely be appropriate in this scenario (rating = 4).

**Tau**

Neurofibrillary tangle burden associated with tau protein deposition correlates more closely with the severity of dementia than amyloid burden does. In a recent large multisite study, tau PET correlated more strongly with longitudinal decline in the Mini-Mental State Examination (MMSE) than amyloid PET did (although less strongly than MRI cortical thickness did) in individuals with amyloid-positive AD dementia (185). Overall, acknowledging the limited available data, the workgroup was somewhat confident that tau PET was appropriate in this scenario (rating = 7).

**Clinical Scenario 14**

**To determine eligibility for treatment with an approved amyloid-targeting therapy**

Consensus ratings

Amyloid - 9 High confidence that use of the tracer is appropriate.

Tau - 8 Moderately confident that use of the tracer is appropriate.

**Amyloid**

Amyloid PET is often used to determine eligibility for enrollment in clinical trials testing anti-amyloid treatment for early AD (224), including the pivotal studies leading to FDA’s accelerated approval of the anti-amyloid monoclonal antibody aducanumab (EMERGE/ENGAGE trials) and full approval of the anti-amyloid monoclonal antibodies lecanemab (CLARITY-AD trial) and donanemab (TRAILBLAZER-ALZ2 trial) for the treatment of MCI and mild dementia due to AD (14–16*,*^225^). In EMERGE, CLARITY-AD, and TRAILBLAZER-ALZ2, treatment with an amyloid-targeting monoclonal antibody was associated with slower cognitive and functional decline compared with that for placebo on primary and secondary clinical endpoints (14–16). The FDA prescribing information and published AURs for aducanumab and lecanemab require biomarker evidence of amyloid pathology (e.g., established via PET or CSF) prior to initiating therapy (226–231). Apart from its high diagnostic accuracy, amyloid PET exhibits some additional advantages over other amyloid biomarkers, such as low variability of the measure across centers and methods (232), low individual variability in healthy subjects, and provision of information on the extent and location of amyloid pathology (53), which may be relevant for selecting candidates for amyloid-targeting therapies. Consequently, the workgroup concluded that amyloid PET is appropriate in patients being evaluated for treatment with approved anti-amyloid therapies (rating = 9). The final rating reflects an increase compared with the original rating in August 2021, which was still in the “appropriate” range (*original rating = 8*).

**Tau**

The use of tau PET in anti-amyloid clinical trials is relatively limited to date. Elevated tau PET was required as an inclusion criterion in the TRAILBLAZER-ALZ2 trial of donanemab (16), and tau PET scans were acquired in a nonrandomized subset of participants in EMERGE/ENGAGE and CLARITY-AD (14–16).

The data available to date suggest that baseline tau PET may predict the magnitude of clinical benefit associated with amyloid removal by monoclonal antibodies. In TRAILBLAZER-ALZ2, clinical outcomes were evaluated separately in a baseline “low-medium” tau PET group and in the “combined population” (16), the latter also including participants with baseline high tau PET. Overall, slowing of clinical decline was greater in the “low-medium” tau group than in the “whole population.” A post hoc analysis suggested limited clinical benefit compared with placebo in patients with “high” tau PET at baseline. An analysis of the tau PET substudy from CLARITY-AD similarly showed that patients with the lowest baseline tau PET derived the greatest clinical benefit from treatment (233). Collectively, the data suggest that amyloid removal may be most clinically beneficial in impaired individuals who are at earlier stages of tau spread as staged by PET. From these data, the workgroup concluded that tau PET is appropriate in patients being evaluated for treatment with approved anti-amyloid therapies (rating = 8). This final rating represents an increase from the initial rating in August 2021, which was in the “uncertain” range (*original rating = 5*). Note that the use of tau PET for treatment eligibility is not included in FDA prescribing information or published AURs for aducanumab, lecanemab, or donanemab (226–231).

**Clinical Scenario 15**

**To monitor response among patients who have received an approved amyloid-targeting therapy**

Consensus ratings

Amyloid - 8 Moderately confident that use of the tracer is appropriate.

Tau - 5 Uncertain, evidence is inconclusive or lacking.

**Amyloid**

Serial amyloid PET scans can be used to measure amyloid plaque removal and thus confirm target engagement in clinical trials of amyloid-lowering therapies that target fibrillar forms of amyloid (14–16*,*^225^*,*^234^–^237^). Conversely, drugs that target soluble forms of amyloid may show slowed accumulation (rather than reductions) of amyloid plaques (238). The FDA determined that lowering of the amyloid PET signal was a suitable surrogate biomarker “reasonably likely to predict a clinical benefit” as a basis for accelerated approval of aducanumab and lecanemab (prior to full approval of the latter based on demonstration of clinical efficacy in phase 3 trials) (14*,*^15^). Further work has suggested that, in the early symptomatic stage of AD, clinical response to amyloid-targeting monoclonal antibodies may be related to the magnitude of plaque reduction, the rapidity of plaque removal, or the ability to suppress amyloid levels below a threshold. All of these outcomes are measured by amyloid PET changes in response to therapy (239–242).

Although in EMERGE/ENGAGE and CLARITY-AD, active antibody treatment was maintained throughout the trials, in TRAILBLAZER-ALZ2 (and its phase 2 predecessor TRAILBLAZER-ALZ), the *duration* of antibody treatment was titrated to amyloid PET response, with patients switched from active treatment to placebo after their amyloid PET scans were in the negative range (16*,*^235^). In both these phase 2 and 3 trials of donanemab, this approach to restricting treatment duration was sufficient to achieve a clinical benefit. From these emerging data, the workgroup felt that measurement of amyloid reduction (e.g., using standardized quantitative methodology such as the CL scale) may be important in guiding management and thus concluded that amyloid PET is appropriate for monitoring response in patients receiving approved amyloid-targeting therapy (rating = 8). This final rating represents an increase from the initial rating in August 2021, which was in the “uncertain” range (*initial rating = 6*). Note that the use of amyloid PET for treatment monitoring is not included in FDA prescribing information or published AURs for aducanumab or lecanemab (226–230), but is included in the donanemab prescribing information (231).

**Tau**

Consistently across trials, amyloid removal by amyloid-targeting monoclonal antibodies led to reductions in fluid (CSF and plasma) measure of P-tau. Data regarding the effects of amyloid removal on tau PET data are more limited and less consistent. In relatively small and nonrandomized subsets of patients enrolled in EMERGE/ENGAGE and CLARITY-AD, amyloid-lowering treatment was associated with reductions or slowed progression of regional tau PET signal (14*,*^15^). In the phase 2 TRAILBLAZER study, amyloid lowering slowed increases in regional (but not global cortical) tau PET (235), but these results were not replicated in the phase 3 TRAILBLAZER-ALZ2 trial (16).

Given that tau PET changes are thought to occur downstream of amyloid and have more established correlations with clinical outcomes, tau imaging has great potential for gauging disease modification in patients treated with anti-amyloid therapies. However, from the limited empirical evidence, the workgroup was uncertain about the appropriateness of tau PET in this scenario (rating = 5). This rating reflects the initial rating in August 2021. Given limited additional data, the workgroup elected *not* to vote again on this scenario in August 2023. Note that use of tau PET for treatment monitoring is not included in FDA prescribing information or published AURs for aducanumab, lecanemab, or donanemab (226–231).

**Clinical Scenario 16**

**Nonmedical usage (e.g., legal, insurance coverage, or employment screening)**

Consensus ratings

Amyloid - 1 Highly confident that the use of the tracer is rarely appropriate.

Tau - 1 Highly confident that the use of the tracer is rarely appropriate.

**Amyloid and Tau**

There is no evidence to suggest that amyloid or tau imaging is more informative than traditional neuropsychological or performance-based assessments to establish the presence, or evaluate the extent, of cognitive or functional impairment. Examples of nonmedical usage include assessments of legal competency, employability, insurability, and fitness to perform activities such as driving, piloting an aircraft, governing, or making financial decisions. The high prevalence of AD pathology in CU older adults further underscores the inappropriateness of amyloid and tau PET for nonmedical purposes. The committee therefore ranked both amyloid and tau PET as “rarely appropriate” in this scenario (rating = 1 for both).

**Clinical Scenario 17**

**In lieu of genotyping for suspected autosomal dominant mutation carriers**

Consensus ratings

Amyloid - 1 Highly confident that the use of the tracer is rarely appropriate.

Tau - 1 Highly confident that the use of the tracer is rarely appropriate.

**Amyloid and Tau**

Dominantly inherited AD (DIAD) is caused by autosomal dominant mutations in the amyloid precursor protein (*APP*), presenilin-1 (*PSEN1*), or presenilin-2 (*PSEN2*) genes. Pedigrees are typically characterized by early onset of symptoms across multiple generations. The standard of care for evaluating potential mutation carriers includes a detailed clinical evaluation, including a family history, and referral to a genetic counselor for discussion of diagnostic or predictive genotyping. Amyloid PET in DIAD becomes positive approximately 2 decades prior to the estimated year of symptom onset (243–246), with cortical binding accompanied in some mutations by early and high binding in the striatum. Rarely, mutations lead to atypical conformations of amyloid (e.g., cotton wool plaques) that do not bind amyloid PET ligands. In contrast, tau PET in DIAD turns positive around the same time that cognitive changes are first detected.

In the future, amyloid and tau PET may be used to evaluate disease stage (i.e., onset and degree of amyloidosis and tau deposition) and will potentially affect decisions about initiating specific therapies. Notably, amyloid-targeting therapies have thus far not been shown to slow cognitive decline in DIAD (224). Moreover, amyloid and tau PET should not be considered alternatives to genotyping, since the absence of a PET signal does not exclude a mutation and, conversely, positive PET scans cannot confirm the presence of DIAD. The workgroup therefore concluded that amyloid and tau PET are rarely appropriate in this scenario (rating = 1 for both).

## 9. VALUE OF TAU PET IMAGING IN COMBINATION WITH AMYLOID PET IMAGING

The current AUC evaluated clinical scenarios for amyloid and tau PET separately for conceptual reasons and clarity and because there was often insufficient evidence to evaluate the combined use of the 2 PET modalities. Although these AUC make no recommendations about the joint use of the 2 PET modalities, considerations of how the 2 complement each other is discussed here. We expect that future investigations will provide an empirical basis for optimizing their joint use.

The markedly different temporal and spatial profiles of amyloid and tau accumulation translates into different relationships between abnormal amyloid and tau PET images for the diagnosis of AD. The specific circumstances will determine which of the 2 PET tracers would be most helpful. Amyloid PET is a more sensitive biomarker for identifying persons who are early in the Alzheimer pathway. Amyloid PET has greater sensitivity in patients with MCI or earlier stages of impairment because tau PET abnormalities in CU persons or those with SCD or MCI are typically absent or very modest. In symptomatic persons, abnormal amyloid PET will not necessarily prove that AD is a relevant etiology if tau PET abnormalities are absent. As the topography of tau PET signal is closely correlated with spatial patterns of AD-related neurodegeneration and domain-specific cognitive performance, a topographically extensive tau PET pattern in a symptomatic person is highly likely to indicate that AD is a relevant etiology. If tau PET abnormalities are absent or spatially limited, the clinician could conclude that other etiologies are likely to be more relevant, even if elevated amyloid by PET is present.

There may be scenarios in which both tracers are required for decision making. In a head-to-head study comparing the clinical utility of amyloid and tau PET, patients were randomized to receive amyloid or tau PET first (and the other modality second) as part of a diagnostic workup (247). Regardless of modality, the first PET scan led to a change in diagnosis in 28% of patients and the second scan changed diagnosis in an additional 18%–19% of patients. The only modality-specific difference found was that a negative amyloid PET scan had a larger impact on diagnosis than a negative tau PET scan did. In another recent study, the addition of tau PET led to a change in diagnosis in 7.5% of memory clinic patients with known amyloid status based on CSF (248). In CU individuals, the combination of positive amyloid and tau PET results is associated with a greatly increased likelihood of conversion to MCI or dementia compared with individuals who have negative results on both modalities, or a positive result on just one (132*,*^133^). As discussed earlier, in the setting of therapeutic interventions targeted at reducing amyloid, it might be necessary to judge the burden of both amyloid and tau initially, as well as to follow both over the course of treatment.

Evolving research and clinical criteria for AD recognize the complementary role of amyloid and tau PET in the diagnosis and staging of AD in living people. In the 2018 NIA-AA Research Framework, PET (and other biomarkers) was used to classify each individual as positive or negative for brain amyloidosis (“A,” e.g., with amyloid PET), tauopathy (“T,” e.g., with tau PET), and neurodegeneration (“N,” e.g., with FDG PET) by using the AT(N) framework (17). In the updated 2024 AA Criteria (18), amyloid PET is considered a “Core 1” biomarker, which is sufficient to establish the diagnosis of AD. Tau PET is considered a “Core 2” biomarker, used to stage disease in patients in whom the diagnosis has already been established with a positive Core 1 biomarker. Using a combination of amyloid and tau PET imaging, Biomarker Stage A is defined by positive amyloid and negative tau PET results; Stage B is defined by positive amyloid PET results and tau PET uptake restricted to the medial temporal lobe; Stage C is defined by positive amyloid PET results and moderate neocortical uptake on tau PET; and Stage D is defined by positive amyloid PET results and high neocortical tau PET uptake. Implementing this staging system in clinical practice will require further refinement and standardization of tau PET clinical and quantitative interpretation methods, compared with the current FDA-approved interpretation method, which requires neocortical tau PET signal and is based solely on visual reads (10).

## 10. LIMITATIONS OF EVIDENCE REVIEW

The outside systematic review of the literature undertaken for this paper was presented more than 3 years prior to publication of these AUC. Since that time, several additional papers evaluating the accuracy and clinical importance of amyloid and tau PET have been published. The authors of these AUC have included these new papers in the bibliography when they were cited in the text; however, these papers were not subject to the same review process and grading as papers included in the initial systematic literature review.

As noted earlier, there are limited data regarding the clinical utility of tau PET in comparison to amyloid PET, in particular pertaining to the impact of each modality on clinical decision making. This difference led to generally higher confidence in the utility of amyloid PET versus tau PET in most clinical scenarios.

Cognitive health disparities, defined here as preventable differences in the prevalence and risk of dementia due to AD and related disorders, are increasingly recognized to disproportionately negatively affect individuals from historically underrepresented racial and ethnic groups. These groups have been markedly underrepresented in AD-related research, including in neuroimaging studies. Limited studies have generally found lower rates of amyloid PET positivity in African-Americans/Blacks, Hispanics/Latinx, and Asian-American Pacific Islanders than in non-Hispanic Whites, ranging from CU research volunteers to patients with MCI and dementia (249–251), although the mechanisms that drive these observed differences are not well understood. Further studies of amyloid and tau PET in underrepresented populations are underway, as are efforts to enhance diversity across longitudinal AD and related disorders research cohorts (252).

Many of the studies comparing amyloid and tau PET to a neuropathological standard-of-truth were conducted in end-of-life patients. Studies validating PET-to-autopsy correlations in more clinically relevant memory clinic populations (i.e., generally younger and less impaired individuals in which imaging would be considered) are needed. There is also increasing recognition that cognitive impairment in older individuals is often related to multiple neuropathologies beyond amyloid and tau (e.g., vascular contributions, Lewy bodies, LATE). More studies are needed to evaluate how co-pathologies affect the clinical interpretation of amyloid and tau PET results.

Finally, published evidence is often based on investigational studies conducted in research settings. When applying such research findings to general clinical patient populations, careful considerations need to be taken, given different pretest probabilities of diseases in various clinical settings and possible inconsistencies in imaging quality, image interpretation accuracy, and other technical factors. It is important to reserve clinical judgments for individual patient considerations and specific clinical settings.

## 11. FURTHER RESEARCH QUESTIONS

Although much progress has been made in the clinical implementation of amyloid and tau PET, there are still many knowledge gaps that should serve as groundwork for future work. With the recent approval of amyloid-targeting monoclonal antibodies, the field has entered a new era of molecular-specific therapies, and amyloid and tau PET are likely to play an increasingly important role in individuals being evaluated for these novel treatments. Beyond their diagnostic value, future work will undoubtedly focus on whether amyloid and tau PET can identify optimal responders to various treatments and whether the duration of treatment can be calibrated on the basis of longitudinal changes in PET. Especially in the context of longitudinal imaging, it will be important to determine whether quantitative approaches to image interpretation enhance the current approach of visual reads. Some data do suggest a combination of visual and quantitative interpretation can improve the accuracy of reads, especially for less experienced nuclear medicine physicians and radiologists (37). PET quantification will likely be essential for gauging response to amyloid-lowering therapies (and possibly in future tau-lowering therapies (47*,*^253^)) in clinical practice and for gauging disease progression. Moving forward, it will be important to collect PET data in patients treated with novel therapies via longitudinal patient registries such as the Alzheimer’s Registry for Treatment and Diagnostics (ALZ-NET) (254). Extraction of CL values from clinically acquired amyloid PET scans has been shown to be feasible (255), and current efforts are underway to standardize tau PET measurements across radiotracers and processing approaches (e.g., the CenTauR scale (44)).

To date, only 1 tau PET tracer (^18^F-FTP) has been approved by the FDA for clinical use, based on a visual read method that highlights neocortical uptake and is insensitive to early-stage (but potentially clinically meaningful) tau pathology (10). PET-to-autopsy studies are currently being conducted with additional tau PET tracers (e.g., ^18^F-MK6240 and ^18^F-PI2620) and using alternative visual interpretation methods, including methods that identify binding that is restricted to the medial temporal lobe (256–258). These studies will determine whether alternative tau tracers or visual interpretation approaches are more sensitive to Braak Stages III/IV, which would affect future clinical recommendations. As noted earlier, augmenting visual reads with semiquantification of the PET signal in clinical practice could also broaden the utility of both amyloid and tau PET in guiding clinical care.

Few studies have evaluated the clinical impact of tau PET on patient diagnosis and management as a single modality or in combination with amyloid PET (247*,*^248^). Future clinical practice guidelines will determine the specific role of PET within the larger landscape of CSF and emerging plasma amyloid and tau biomarkers. Although much of the initial work on clinical utility has focused on diagnosis and patient management, data are beginning to emerge regarding the impact of amyloid PET on longer term health outcomes, including inpatient and outpatient resource utilization, institutionalization, and even mortality (259*,*^260^). Finally, acknowledging the transformative impact of amyloid and tau PET on AD research and drug development, there remains a huge unmet need to develop molecular imaging markers for other protein aggregates, such as non-AD tauopathies, α-synuclein, and TDP-43, to truly capture the complexity of brain pathologies that contribute to neurodegeneration and dementia (see Appendix C).

## APPENDIX ABBREVIATIONS

AA: Alzheimer’s Association
Aβ: Amyloid beta
AD: Alzheimer’s disease
ADCS-PACC: Alzheimer Disease Cooperative Study– Preclinical Alzheimer’s Cognitive Composite
ADNC: Alzheimer’s disease neuropathological changes
ADNI-1: Alzheimer’s Disease Neuroimaging Initiative initial phase
*APOE4*: Apolipoprotein E ε4 allele
*APP*: Amyloid precursor protein gene
AUC: Appropriate use criteria
AUR: Appropriate use recommendation
CBD: Corticobasal degeneration
CBS: Corticobasal syndrome
CDR: Clinical dementia rating
CDR-G: Clinical dementia rating-global
CERAD: Consortium to Establish a Registry for Alzheimer’s Disease
CL: Centiloids
CMS: Centers for Medicare and Medicaid Services
CN: Cognitively normal
COI: Conflict of interest
CSF: Cerebrospinal fluid
CU: Cognitively unimpaired
DAT: Dementia of the Alzheimer type
DIAD: Dominantly inherited Alzheimer’s disease
DLB: Dementia with Lewy bodies
EANM: European Association of Nuclear Medicine
FDA: Food and Drug Administration
FDG: Fluorodeoxyglucose
FTD: Frontotemporal dementia
FTLD: Frontotemporal lobar degeneration
FTP: Flortaucipir
GRADE: Grading of recommendations, assessment, development, and evaluations
HC: Healthy controls
IDEAS: Imaging Dementia—Evidence for Amyloid Scanning
IHC: Immunohistochemical
KQ: Key question
LATE: Limbic-predominant age-related TDP-43 encephalopathy
lvPPA: Logopenic-variant of primary progressive aphasia
MCI: Mild cognitive impairment
MCI-LB: MCI with Lewy antibodies
MMSE: Mini-mental state examination
MRI: Magnetic resonance imaging
MTBR-tau243: Microtubule-binding region of tau at residue 243
N/A: Not available
NFTs: Neurofibrillary tangles
NIA-AA: National Institute on Aging and Alzheimer’s Association
NINCDS-ADRDA: National Institute of Neurological and Communicative Disorders and Stroke and the Alzheimer’s Disease and Related Disorders Association
NR: Not reported
OHSU: Oregon Health & Science University
PACC: Preclinical Alzheimer’s Cognitive Composite
PCA: Posterior cortical atrophy
PDD: Parkinson’s disease with dementia
PET: Positron emission tomography
PiB: Pittsburgh compound-B
PICOTS: Population, interventions, comparisons, outcomes, timing, and settings
*PSEN1*: Presenilin-1 gene
*PSEN2*: Presenilin-2 gene
PSP: Progressive supranuclear palsy
P-tau: Phosphorylated tau
RCT: Randomized controlled trial
REM: Rapid eye movement
SCC: Subjective cognitive complaints
SCD: Subjective cognitive decline
SCI: Subjective cognitive impairment
SMD: Subjective memory decline
SNAP: Suspected non-Alzheimer’s pathophysiology
SNMMI: Society of Nuclear Medicine and Molecular Imaging
SPECT: Single photon emission computed tomography
SUVR: Standardized uptake value ratio
TDP-43: TAR DNA-binding protein 43

### DISCLOSURES AND CONFLICTS OF INTEREST

The AA, SNMMI, and Avalere rigorously attempted to avoid any actual, perceived, or potential conflicts of interest (COIs) that might have arisen because of an outside relationship or personal interest of workgroup members. Both organizations reviewed their own industry relationship policies to ensure that the ensuing process adhered to both standards.

**Table:** Relationships with Industry and Other Entities

Workgroup member	Conflict of interest
Javier Arbizu, MD, PhD	Clinical research for Araclon Biotech; institution received research support from Life Molecular Imaging; served as a consultant for Eli Lilly
Tammie L.S. Benzinger, MD, PhD	Consultant for Lilly, Biogen, Eisai, and Johnson & Johnson; investigator initiated research funded by Siemens
Kevin Donohoe, MD	The author declares that there is no conflict of interest
Oskar Hansson, MD, PhD	Institution received research support from ADx, AVID Radiopharmaceuticals, Biogen, Eli Lilly, Eisai, Fujirebio, GE Healthcare, Pfizer, and Roche; in the past 2 y has received consultancy/speaker fees from AC Immune, Amylyx, Alzpath, BioArctic, Biogen, Bristol Meyer Squibb, Cerveau, Eisai, Eli Lilly, Fujirebio, Merck, Novartis, Novo Nordisk, Roche, Sanofi, and Siemens
Peter Herscovitch, MD	Associate Editor for Sage Publishing
Keith Johnson, MD	Clinical trial for Cerveau Technologies and consultant for Novartis, Genentech, Jansson, Takeda, Merck, and Prothena
David Knopman, MD	The author declares that there is no conflict of interest
Phillip H. Kuo, MD, PhD	Consultant or speaker for Blue Earth Diagnostics, Chimerix, Eli Lilly, Fusion Pharma, General Electric Health Care, Invicro, Novartis, Radionetics, and Telix Pharmaceuticals; recipient of research grants from Blue Earth Diagnostics and General Electric Health Care
Jennifer Hagerty Lingler, PhD	Consultant to Biogen and Genentech and has received research support from Avid Radiopharmaceuticals, a wholly owned subsidiary of Eli Lilly
Satoshi Minoshima, MD, PhD	Consultant and received educational donation from Hamamatsu Photonics, research grant from Hitachi, and education donation from Nihon Medi-Physics Co., Ltd.
Melissa E. Murray, PhD	Consulted for AVID Radiopharmaceutical and receives research support from Eli Lilly
Julie C. Price, PhD	The author declares that there is no conflict of interest.
Gil Rabinovici, MD	Institution received research support from Avid Radiopharmaceuticals, GE Healthcare, Life Molecular Imaging, and Genentech; served as a consultant for Eli Lilly, Johnson & Johnson, and Merck
Stephen Salloway, MD, MS	Institution received research support for clinical trials from Biogen, Janssen, Eisai, Lilly, Genentech, and Roche; served as a consultant for Merck, Novo Nordisk, and Acumen
Christopher J. Weber, PhD	Full-time employee of the Alzheimer’s Association. No financial conflicts to disclose.
Maria C. Carrillo, PhD	Full-time employee of the Alzheimer’s Association and has a daughter in the neuroscience program at USC; no financial conflicts to disclose

The workgroup members were required to provide disclosure statements of all relationships that might be perceived as a real or potential COI. These statements were reviewed and discussed by the workgroup cochairs and updated and reviewed by an objective third party at the beginning of every task force meeting and/or teleconference. Disclosures for task force members can be found in Table 4.

To adjudicate the COIs, the leadership from the AA, SNMMI, and Avalere first determined the threshold for a real COI. Following consultation with various experts and review of other policies used, the team defined COIs as the following: An individual that had relationships with industry, including consulting, speaking, research, and other non-research activities, that exceed $5,000 in funding over the previous or upcoming 12-mo period.
